# Compartmentalized Replication of R5 T Cell-Tropic HIV-1 in the Central Nervous System Early in the Course of Infection

**DOI:** 10.1371/journal.ppat.1004720

**Published:** 2015-03-26

**Authors:** Christa Buckheit Sturdevant, Sarah B. Joseph, Gretja Schnell, Richard W. Price, Ronald Swanstrom, Serena Spudich

**Affiliations:** 1 Department of Microbiology and Immunology, University of North Carolina at Chapel Hill, Chapel Hill, North Carolina, United States of America; 2 Lineberger Comprehensive Cancer Center, University of North Carolina at Chapel Hill, Chapel Hill, North Carolina, United States of America; 3 Department of Neurology, University of California, San Francisco, San Francisco, California, United States of America; 4 UNC Center for AIDS Research, University of North Carolina at Chapel Hill, Chapel Hill, North Carolina, United States of America; 5 Department of Biochemistry and Biophysics, University of North Carolina at Chapel Hill, Chapel Hill, North Carolina, United States of America; 6 Department of Neurology, Yale University School of Medicine, New Haven, Connecticut, United States of America; Frederick National Laboratory for Cancer Research, UNITED STATES

## Abstract

Compartmentalized HIV-1 replication within the central nervous system (CNS) likely provides a foundation for neurocognitive impairment and a potentially important tissue reservoir. The timing of emergence and character of this local CNS replication has not been defined in a population of subjects. We examined the frequency of elevated cerebrospinal fluid (CSF) HIV-1 RNA concentration, the nature of CSF viral populations compared to the blood, and the presence of a cellular inflammatory response (with the potential to bring infected cells into the CNS) using paired CSF and blood samples obtained over the first two years of infection from 72 ART-naïve subjects. Using single genome amplification (SGA) and phylodynamics analysis of full-length *env* sequences, we compared CSF and blood viral populations in 33 of the 72 subjects. Independent HIV-1 replication in the CNS (compartmentalization) was detected in 20% of sample pairs analyzed by SGA, or 7% of all sample pairs, and was exclusively observed after four months of infection. In subjects with longitudinal sampling, 30% showed evidence of CNS viral replication or pleocytosis/inflammation in at least one time point, and in approximately 16% of subjects we observed evolving CSF/CNS compartmentalized viral replication and/or a marked CSF inflammatory response at multiple time points suggesting an ongoing or recurrent impact of the infection in the CNS. Two subjects had one of two transmitted lineages (or their recombinant) largely sequestered within the CNS shortly after transmission, indicating an additional mechanism for establishing early CNS replication. Transmitted variants were R5 T cell-tropic. Overall, examination of the relationships between CSF viral populations, blood and CSF HIV-1 RNA concentrations, and inflammatory responses suggested four distinct states of viral population dynamics, with associated mechanisms of local viral replication and the early influx of virus into the CNS. This study considerably enhances the generalizability of our results and greatly expands our knowledge of the early interactions of HIV-1 in the CNS.

## Author Summary

Early HIV-1 CNS replication likely provides a foundation for brain injury and a potentially important tissue reservoir. To explore the character and timing of emergence of early HIV-1 CNS replication, we examined paired cerebrospinal fluid (CSF) and blood samples from 72 ART-naïve adults, with one-half having longitudinal samples, during the first two years following HIV-1 subtype B infection. In a cross sectional analysis over the first two years of infection, 10–25% of subjects had evidence of either local viral replication in the CNS, defined by the presence of CSF compartmentalization, or a robust inflammatory response, and in approximately 16% of subjects this CNS involvement persisted over time. In some subjects, one of two transmitted viruses replicated predominantly within the CNS, providing insight into how HIV-1 can establish independently replicating populations early in different parts of the body. Based on their entry phenotype, all viruses were selected for replication in CD4+ T cells, although this phenotype was slightly altered in the compartmentalized virus. Overall, we suggest four states to model the nature of HIV-1 CNS infection, which imply distinct mechanisms of virus/host interaction within the CNS during early infection.

## Introduction

While HIV-1 can be detected in both the cerebrospinal fluid (CSF) and brain tissue during the weeks after initial exposure [1–7], it is unknown when the virus actually begins replicating independently in the central nervous system (CNS). Independent viral replication within the CNS has two important implications. First, HIV-1 replication can lead to CNS dysfunction and injury, and while combination antiretroviral therapy (cART) has markedly reduced the incidence of HIV-associated dementia (HAD), the prevalence of milder HIV-associated neurological disorders (HAND) has increased [8,9] in the cART era. Second, independent CNS replication may also provide a reservoir distinct from that found in CD4+ T cells in the blood and lymphoid tissue. We do not know the time course of the virologic events that lead to neurological dysfunction and the potential establishment of a CNS reservoir, or the extent to which these long-term outcomes are predicted by the initial aspects of virus-host interaction.

While extensive independent, or compartmentalized, CSF/CNS replication is associated with severe HIV-1 clinical CNS dysfunction [1,10–13], genetically distinct virus can be detected in the CNS throughout the course of infection [4,10]. Thus far, two types of compartmentalization have been defined: one in which a few variants are rapidly expanded giving a CSF viral population of low complexity (clonal amplification) consisting of variants that require high levels of CD4 for entry (R5 T cell-tropic). The second type is characterized by a complex CSF viral population consisting of variants that can enter cells expressing low levels of CD4 (macrophage-tropic), indicative of a more prolonged period of isolated replication and evolution of the entry phenotype. Additionally, we have recently shown that after vertical transmission to children, CNS compartmentalization can be established via two mechanisms: the early sequestration of one of multiple transmitted variants in the CNS, or the later establishment of compartmentalized CNS virus originating from the periphery [14]. In a previous study, we demonstrated CSF HIV-1 compartmentalization in human subjects during the first year of HIV-1 infection in adults [4]. However, that study only examined viral population sequences from CSF and plasma in a small number of subjects (eight), limiting the generalizability of our findings. Furthermore, the previous study had sparse assessment of longitudinal samples, and included no analysis of sources of compartmentalized HIV-1 within the CSF.

For the current study, we used single genome amplification (SGA) and phylogenetic analysis to assess viral populations in the CSF in the presence and absence of cellular inflammation (i.e. pleocytosis) in cross-sectional and often longitudinal paired blood plasma and CSF samples obtained during the first two years of HIV-infection in ART-naïve subjects. We also extended our analytical approach to include Bayesian Evolutionary Analysis by Sampling Trees (BEAST) to assess time to most recent common ancestor (TMRCA) of CSF and blood HIV-1 populations [15], providing new insights on the timing of establishment of early compartmentalized populations, and we assessed the entry phenotypes of selected clones, further confirming the nature of the transmitted virus as R5 T cell-tropic [16]. Based upon a complex interplay between HIV-1 RNA concentration, viral compartmentalization, and CSF white blood cell (WBC) count, we suggest at least four different patterns to characterize the relationship between virus in the blood and virus in the CSF/CNS during the early period after infection. The current study considerably enhances the generalizability of our results and provides an unprecedented view of the early interactions of HIV-1 in the CNS.

## Results

### Study population and analysis parameters

We analyzed the HIV-1 RNA concentrations in paired blood plasma and CSF samples collected from 72 adult subjects enrolled in an observational neurological study of primary HIV-1 infection, defined as within one year of initial infection. All subjects were infected with HIV-1 subtype B and were ART-naïve at all study intervals, except for one subject, 9018, who was treated with tenofovir, emtricitabine, and atazanavir between the first and second analyzed time points. Paired follow-up samples were assessed for 37 subjects with longitudinal samples available within the initial two years post infection (p.i.). In total, 144 paired samples were available for analysis. Baseline demographic and clinical characteristics at enrollment for the entire cohort (n = 72) and for the subset that had sufficient CSF viral RNA concentrations (defined as greater than 1,000 copies of viral RNA/ml) to allow adequate sampling of the viral population via SGA (n = 33) are shown in Table 1. For the 33 subjects whose samples were analyzed by SGA, a total of 55 blood plasma/CSF sample pairs were analyzed including the longitudinal samples (Table 2; three time points for subject 9018 and one time point for subject 9040 beyond 2 years p.i. were analyzed but were not included in any overall population analysis.)

**Table 1:** Background demographic and clinical characteristics of study participants at baseline.

	Entire PHI cohort (n = 72)	Subjects analyzed by SGA (n = 33)^a^
Sex, % male	98.6	97.0
Age (years)	36 (28–43)	36 (31–44)
Days post infection	106 (73–158)	128 (76–173)
CD4+ T cells (cells/μl)	567 (411–732)	488 (361–714)
plasma HIV-1 RNA (log10 copies/ml)	4.66 (4.09–5.08)	5.06 (4.72–5.58)
CSF HIV-1 RNA (log10 copies/ml)	2.62 (1.74–3.32)	3.37 (2.97–3.82)
CSF WBC (cells/μl)	6.0 (3.0–10.8)	7.0 (4.0–12.0)
CSF/plasma albumin ratio	5.0 (4.0–6.8)	5.2 (3.7–6.8)

Values shown are medians (interquartile ranges) except where noted

^a^Subjects with sufficient viral RNA loads in the CSF (>1,000 copies of viral RNA/ml; to ensure adequate sampling) for further SGA analysis at one or more time points within the first two years.

**Table 2:** Subject population virologic, clinical and phylogenetic characteristics.

							**Analyses of compartmentalization** ^**h**^					
							**SM**	**F_ST_**	**S_nn_**			
**Subject ID** ^**a**^	**Days p.i.** ^**b**^	**VL Plasma** ^**c**^	**VL CSF** ^**d**^	**CD4** ^**e**^	**CSF WBC** ^**f**^	**Albumin ratio** ^**g**^	**P Value**	**P Value**	**P Value**	**CNS Compart** ^**i**^	**TMRCA (days)** ^**j**^	**Comp TMRCA (days)** ^**k**^
7146	156	5.63	5.23	552	86	14.35	0.021	0.013	0.005	Cp, Ap	965*	85
	177	5.34	4.4	476	97	14.35	0.1599	0.0157	0.313	Eq		
	203	5.01	4.41	310	53	7.12	0.0058	<0.0001	<0.0001	Cp, Ap		
9001	308	5.29	3.07	573	4	3.5	0.4296	0.05658	0.2458	Eq	378	
	399	4.87	3.61	575	5	4.33	0.83	0.1454	0.1252	Eq	456	
9002	275	4.65	3.66	539	8	7.35	0.59	0.1843	0.1346	Eq	296	43
	338	4.57	4.08	501	14	9.61	0.3192	0.0334	0.666	Eq, Ap	309	76
9003	375	4.56	3.18	521	4	3.19	0.8633	0.8188	0.3676	Eq	1292*	
9006	153	4.44	3.11	435	8	4.64	0.5726	0.4418	0.2694	Eq	167	
	340	4.82	3.21	453	6	3.75	0.8377	0.487	0.3086	Eq	493	
9007	149	5.37	2.63	256	4	10.85	0.6991	0.0946	0.5982	Eq	307	
	406	4.47	3.59	292	4	6.87	0.1191	0.0614	0.0036	Eq	231	117
9015	77	5.41	3.33	474	6	7.4	0.6269	0.7226	0.811	Eq	153	
9016	409	5.06	3.38	344	0	4.2	0.4389	0.4254	0.388	Eq	390	
	691	4.94	3.12	237	5	4.3	0.5313	0.3026	0.322	Eq	592	
9018	200	5.61	4.57	350	11	6.45	0.2295	0.0008	0.1166	Eq, Ap	2277*	140
	687	4.8	4.47	491	119^l^	9.29	0.4681	0.5672	0.2728	Eq		
	876	4.65	4.55	425	71	11.84	0.3772	0.1312	0.2978	Eq		
	1073	4.95	3.99	453	14	7.97	0.0631	0.0026	0.0456	Eq, Ap		
	1180	4.98	4.61	505	17	5.34	<0.0001	<0.0001	<0.0001	Cp, Ap		
9019	100	4.69	3.14	750	13	5.58	0.0956	0.6085	0.0026	Eq	82	
9020	240	4.59	3.45	368	10	6.95	0.0049	0.0076	0.0024	Cp	608*	246
9021	140	4.82	3.37	441	8	4.89	0.0017	0.0008	0.0068	Cp, Ap	159	102
	341	5.8	4.01	446	71	5.21	0.0021	<0.0001	0.0006	Cp, Ap	314	55
9024	222	4.75	4.29	974	12	5.74	0.913	0.2318	0.0412	Eq	196	
	405	4.63	3.73	732	5	4.34	0.6906	0.3016	0.3252	Eq	379	
	596	4.4	4.01	600	10	5.58	0.7406	0.2084	0.5158	Eq	1045	
9025	50	5	4.18	752	7	3.91	0.0743	0.3496	0.098	Eq	49	
9027	234	4.84	3	402	6	6.17	0.3348	0.134	0.7062	Eq	236	115
	425	4.76	3.39	375	5	6.11	0.9693	0.984	0.6206	Eq	775	
9036	223	4.48	3.11	610	4	6.07	0.3106	0.5858	0.0552	Eq	386	
9037	46	5.71	3.43	884	26	6.35	0.4384	0.1944	0.4818	Eq	52	
9039	36	5.57	4.3	539	53	9.31	0.4768	0.0136	0.4114	Eq	89	
9040	165	4.22	3.79	705	4	7.67	<0.0001	<0.0001	<0.0001	Cp, Ap	209	33
	352	4.88	3.15	664	3	9.19	<0.0001	<0.0001	<0.0001	Cp, Ap	262	131
	644	5.34	3.64	484	2	8.15	0.002	0.0006	<0.0001	Cp	553	321
	918	5.44	3.37	279	4	8.33	0.3973	0.0546	0.324	Eq		
9044	128	4.82	3.49	533	20	4.3	0.4608	0.0482	0.199	Eq, Ap	124	50
	190	5.08	3.98	583	30	4.01	0.7711	0.6476	0.2786	Eq, Ap	307	
9045	54	4.75	3.1	542	9	4.46	0.2913	0.36	0.125	Eq, Ap	214	
9048	58	5.21	3.1	456	11	7.52	0.4808	0.6226	0.3934	Eq	131	73
9055	106	5.38	3.51	619	12	6.42	0.0239	0.0576	0.2396	Eq	83	
9058	110	5.18	3.66	237	4	2.44	0.2438	0.4112	0.1512	Eq	398	
9062	103	4.88	3.43	420	16	4.09	0.4486	0.9792	0.462	Eq	89	
9063	86	5.06	3.01	558	4	3.38	0.3342	0.3428	0.5326	Eq	565*	
9071	128	5.59	3.93	279	6	4.31	0.8112	0.6726	0.7572	Eq	124	
	177	5.64	4.08	468	13	4.3	0.9343	0.828	0.9708	Eq	147	
9073	75	5.68	4.01	297	42	2.07	0.9946	0.9484	0.9906	Eq	765*	
9076	185	5.66	3.85	111	4	4.09	0.9866	0.597	0.931	Eq	257	
9082	106	4.9	3.94	421	32	2.14	0.99329	0.316	0.4412	Eq	91	
9083	151	5.37	3.72	352	7	3.55	0.9428	0.6502	0.2848	Eq	374	
	344	5.31	3.55	389	3	5.48	0.1948	0.2918	0.7494	Eq	754	
9096	271	5.58	3.77	666	3	2.68	0.0915	0.0376	0.008	Eq	649	253
	348	4.39	3.53	743	9	3.02	<0.0001	<0.0001	<0.0001	Cp, Ap	707	337
9097	55	4.9	3.29	802	8	11.95	0.0163	0.0016	0.0394	Eq^m^	63	

^a^Time point(s) beyond 2 years p.i. analyzed for subject 9018 and 9040 were not included in any overall population analyses.

^b^Estimated.

^c,d^VL, viral load; HIV-1 RNA (log_10_ copies/ml).

^e^Cells/μl.

^f^CSF white blood cell (WBC) count, cell/μl.

^g^CSF/plasma albumin ratio.

^h^Three statistical analyses of genetic compartmentalization between viral populations in the blood plasma and CSF: Slatkin-Maddison test (SM) [18], Wright’s measure of population subdivision (F_st_) [19,20] and the Nearest-neighbor statistic (S_nn_) [21]. *P* values <0.05 indicated statistically significant genetic compartmentalization.

^i^HIV-1 population characteristics in the CSF compartment (compart). Eq, equilibrated blood plasma and CSF populations; Cp (compartmentalized), significant compartmentalization by three compartmentalization analyses; Ap, clonal amplification of ≥3 variants detected in the CSF.[18]

^j^TMRCA (Time to Most Recent Common Ancestor) of the entire viral population, analyzed by BEAST [15]. An asterisk (*) indicates transmission of > 1 variant.

^k^TMRCA for the compartmentalized (Comp) CSF population for compartmentalized subjects.

^l^Patient 9018 diagnosed with neurosyphilis at indicated date.

^m^Significant compartmentalization scores were due to a compartmentalized lineage in the plasma. After removing this plasma lineage, the remaining plasma and CSF sequences were equilibrated.

**Fig 1:**
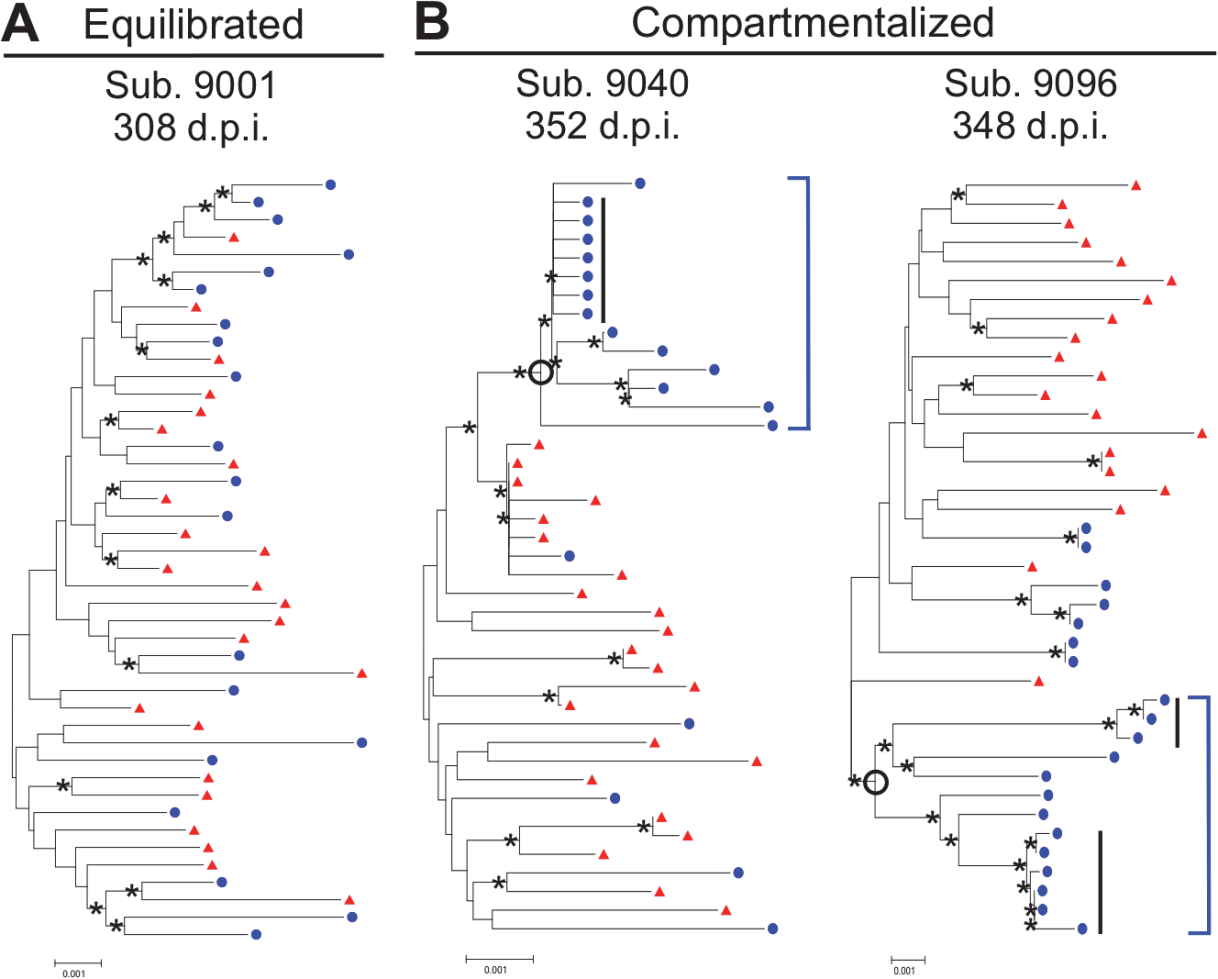
HIV-1 blood and CSF populations early during infection can be equilibrated or compartmentalized. Neighbor-joining phylogenetic trees depicting sequence relationships from subjects with (A) equilibrated and (B) compartmentalized viral populations. *env s*equences from the CSF (blue circles) and blood plasma (red triangles) are shown. Bootstrap values ≥35 are included (*) at the appropriate nodes. Genetic distance is indicated at the bottom of each figure (0.001, number of nucleotide substitutions per site between *env* sequences). Compartmentalized CNS populations are designated by an open circle at the appropriate node and a solid blue bracket. Clonal amplification is indicated by a solid black bar.

Following SGA and phylogenetic analysis, compartmentalization was assessed by three approaches. The choice to use multiple approaches was based on the recent findings of Zarate *et al*. [17] illustrating that different methods of assessing compartmentalization often yield divergent results. Thus, we assessed CNS compartmentalization using three methods—the tree-based Slatkin-Maddison test (SM) [18] and two distance-based methods, Wright’s measure of population subdivision (F_st_) [19,20] and the Nearest-neighbor statistic (S_nn_) [21]. CNS lineages were interpreted as being significantly compartmentalized if all three tests were significant (*P* values < 0.05). We define CNS phylogenetic states as (Fig. 1): i) *equilibrated*, with similar populations between the blood and CSF, with no evidence of independent CNS replication (Fig. 1A); and ii) *compartmentalized*, with a genetically distinct CSF population as indicated by three statistically significant measures of compartmentalization (Fig. 1B). In our analyses, compartmentalized populations typically included clonally amplified variants often with more genetically diverse variants (Fig. 1B, Sub. 9040), and sometimes with the presence of recombinants between two clonally amplified variants (Fig. 1B, Sub. 9096). In order to determine how often our statistical assessment of CNS compartmentalization was due to the presence of clonally amplified viruses, we performed additional analyses in which we collapsed clonally amplified sequences into a single sequence and repeated the tests of compartmentalization. We determined that of the eight sample pairs that displayed both clonal amplification and CNS compartmentalization, three were significantly compartmentalized after collapsing the clonal sequences. This suggests that clonal amplification may drive the statistical assessment of compartmentalization or may be a symptom of ongoing CNS replication. Under this latter scenario, we propose that ongoing viral replication in the CNS may produce diverse, CNS-specific lineages and trigger an influx of T cells that amplify some of these lineages. Table 2 shows the clinical and virologic assessment for each subject who had at least one time point analyzed by SGA, and S1 Table shows this information for the 39 subjects who had no time points analyzed by SGA.

We also considered what role cellular inflammation (pleocytosis) might play in determining both the HIV-1 RNA concentrations and the nature of the viral population in the CSF, specifically whether an equilibrated population might exist with higher CSF HIV-1 RNA concentrations due to an influx of cells, including infected cells, during an inflammatory response. For our analyses, we chose a cut-off of 10 WBC/μl to define a state of CSF pleocytosis, as this is two-times the published upper limit of normal values of CSF WBC (5 cells/μl) [22] ensuring that the measured pleocytosis was a robust marker for an inflammatory response.

### Approximately two-thirds of subjects have low CSF viral RNA concentrations during the first two years of infection

**Fig 2:**
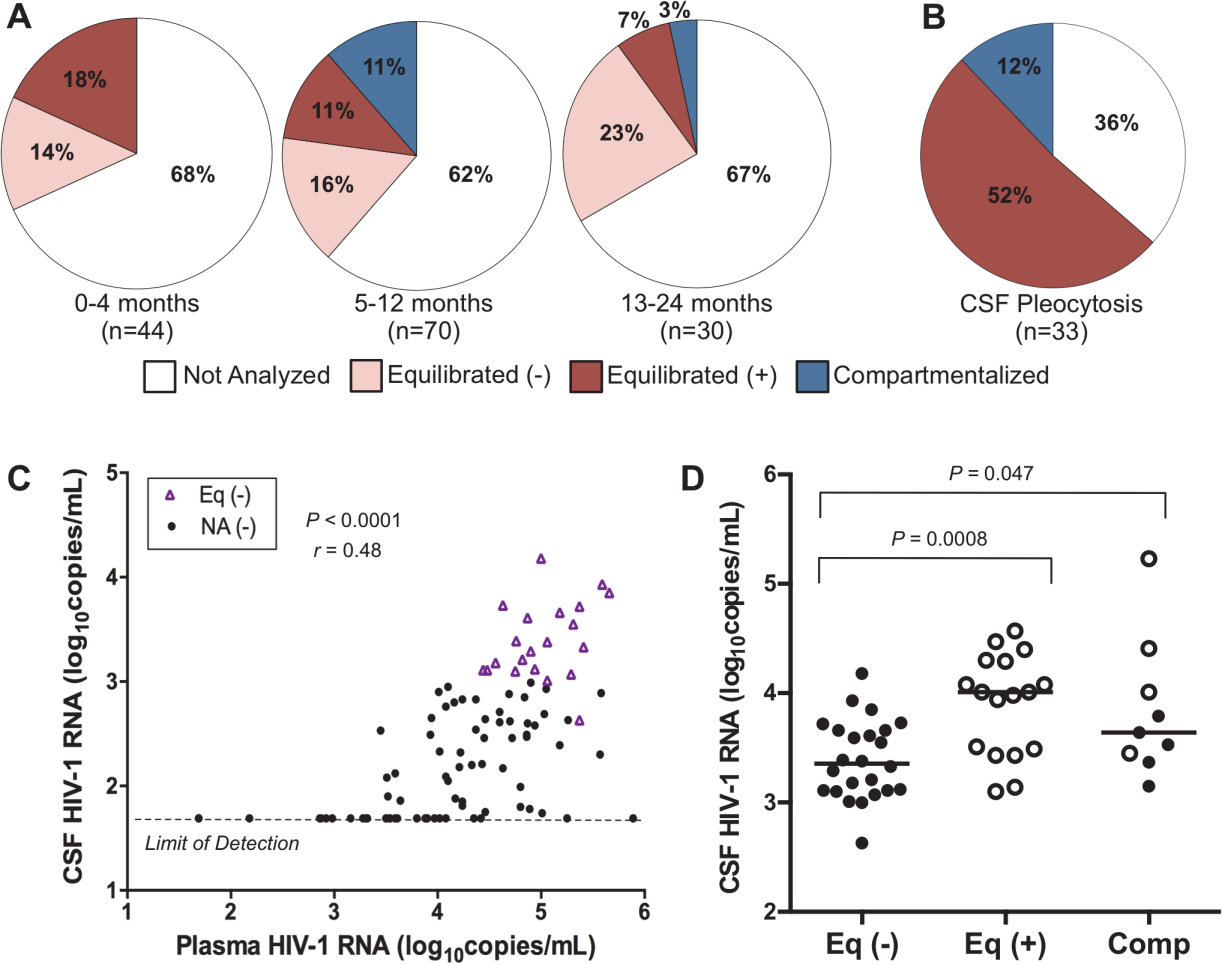
Correlates of CSF HIV-1 RNA levels, CNS inflammation, and CNS phylogenetic state. (A) Pie charts grouping the samples by days p.i. and showing the percentage of samples in each phylogenetic state as a function of time: 0–4 months (‘acute’), 5–12 months (‘early’), and 13–24 (‘established’). States represented include: Not Analyzed by SGA, due to CSF viral load <1,000 copies/ml; Equilibrated (−), CSF WBC <10 cells/μl; Equilibrated (+), CSF WBC ≥10 cells/μl; and Compartmentalized. We used a cut-off of 10 WBC/μl to define a state of substantial CSF pleocytosis because this is two-times the upper limit of normal value of CSF WBC (0 to 5 cells/μl) [22] ensuring that the measured pleocytosis was a robust marker for an inflammatory response. (B) Pie chart showing the percent of samples in each phylogenetic state exhibiting substantial CSF pleocytosis (CSF WBC ≥10 cells/μl). (C) Relationship between plasma and CSF HIV-1 RNA concentrations for not analyzed samples with minimal pleocytosis (NA (−); CSF WBC <10 cells/μl) and equilibrated samples with minimal pleocytosis (Eq (−), CSF WBC <10 cells/μl). Spearman’s rank correlation coefficient and *P*-value are indicated. Samples with undetectable CSF HIV-1 RNA (<50 copies/ml; limit of detection) were not included in the correlation analysis. (D) Relationship between CSF viral loads for equilibrated with minimal pleocytosis (Eq (−); CSF WBC <10 cells/μl); equilibrated with marked pleocytosis (Eq (+); CSF WBC ≥10 cells/μl); and compartmentalized (Comp) samples. Significant *P* values (Mann-Whitney Test) comparing relationships between groups are indicated. Samples with evidence of marked pleocytosis (CSF WBC ≥10 cells/μl) are indicated using open circles, while samples with minimal to no pleocytosis are indicated by solid circles.

In order to assess temporal patterns of CNS viral replication and inflammation, we treated all 144 paired samples from the 72 subjects as independent observations (a limitation of the analysis but one that allowed us to categorize the samples by time post infection). We divided the samples into the following three windows: acute, 0–4 months p.i.; early, 5–12 months p.i.; or established, 13–24 months p.i.. The choice of these times also allowed us to bin the data into groups with reasonable sample sizes. Regardless of length of time since HIV-1 infection, the CSF viral RNA concentration was less than 1,000 copies/ml in approximately two-thirds of samples (Fig. 2A), suggestive of little local production of virus in the CNS, at least as assessed by viral load. Analysis of the viral populations in the remaining samples showed that in approximately 30% of the paired samples the sequence composition of the viral population in the CSF was similar to the viral population in the blood (i.e. equilibrated), and pleocytosis was detected in the CSF of one-quarter to one-half of the samples with these equilibrated populations (Fig. 2A). Compartmentalized viral populations were also detected but exclusively after the first 4 months, in 11% of samples in the early group (total n = 70) and 3% of the established group (total n = 30). However, those samples in which pleocytosis was detected were significantly less likely to have viral RNA levels in the CSF of less than 1,000 copies/ml compared to the total group of samples (see below). The percentages of samples in each phylogenetic group with pleocytosis (Fig. 2B) and without pleocytosis are noted (S1 Fig.).

We next assessed factors associated with the CSF viral load. We first examined subjects without evidence of inflammation (i.e. no pleocytosis) and either low viral load in the CSF (less than 1,000 copies/ml) or an equilibrated population as evidence of no sustained local replication. In these subjects the HIV-1 RNA concentration in the CSF was proportionally 1–2% of the level in the blood (Spearman’s rank correlation coefficient = 0.48, *P*<0.0001) (Fig. 2C). We could not determine whether this proportional relationship was maintained for those samples in which the CSF viral load was undetectable (<50 copies/ml), although there was a trend for these samples to be from subjects with low plasma HIV-1 RNA concentrations (Fig. 2C). We hypothesize that this low level of virus in the CSF is due to the normal trafficking of T cells into the CNS, including infected CD4+ T cells that release the observed virus.

### Pleocytosis can be associated with elevated viral load in the CSF and an influx of virus from the blood

Pleocytosis was detected in 36% of all subjects (Table 2 and S1). Consistent with our prior findings in HIV-infected subjects, lymphocytes were the predominant cell type in subjects with and without elevated levels of cellular inflammation [23]. When pleocytosis was detected, the HIV-1 RNA concentration was twice as likely (64%) to be above 1,000 copies/ml in the CSF compared to the overall population of which two-thirds were below 1,000 copies/ml. Additionally, when pleocytosis was present and the CSF viral load was high enough for SGA analysis, the viral populations were most often equilibrated (Fig. 2D, open circles), with a CSF viral load that was significantly higher than for equilibrated populations without pleocytosis (*P* = 0.0008, Mann-Whitney Test) (Fig. 2D). This suggests that the influx of infected CD4+ T cells associated with pleocytosis brings virus into the CSF/CNS from the blood. There was a trend toward increased CSF:blood albumin ratio (a marker for reduced blood-brain barrier integrity) in the presence of pleocytosis, which could contribute to an influx of virus (Table 2). However, there was a similar (and statistically significant) increase in CSF:blood albumin ratio in the compartmentalized subjects that did not account for the increase in CSF HIV-1 RNA concentration compared to the samples with equilibrated populations without pleocytosis (Fig. 2D; see below), which was instead due to local virus production. These data suggest that increased viral burden in the CNS can result from two factors: independent CNS replication, generating compartmentalized CSF populations, or an influx of infected cells as the result of the inflammatory response of pleocytosis, producing a viral population in the CSF that is similar to that in the blood. It is possible that much of the pleocytosis seen in these subjects is in response to HIV-1 replication in the CNS where the influx of infected cells from the blood produces elevated levels of virus that obscure the detection of a lower level of the locally produced and compartmentalized virus responsible for inducing the inflammation. Other agents that induce pleocytosis should have a similar effect on viral load in the CSF and this was seen in an incident of neurosyphilis in subject 9018 when sampled at day 687 p.i. (Table 2). However, we note that pleocytosis is not always associated with higher viral load (Table 2 and S1), indicating a more complex and perhaps dynamic relationship between pleocytosis and viral load.

### Compartmentalized viral populations early are associated with higher CSF viral loads, and local replication and/or inflammation can persist or reoccur over time

Little evidence for local CNS replication was detected until after 4 months of infection (Fig. 2A), suggesting that detectable CNS-compartmentalized populations are present at greater frequency with a longer time since HIV-1 exposure. As noted above, CSF samples with compartmentalized populations had elevated viral RNA loads when compared to the samples with only equilibrated populations in the absence of pleocytosis (Fig. 2D), consistent with local production of virus with the detection of compartmentalization.

We used cross sectional analyses to examine when these markers of local replication and inflammation were observed. We estimated the percentage of samples with no evidence of viral involvement in the CNS from the number with a CSF viral RNA concentration less than 1,000 copies/ml (Fig. 2A; unanalyzed samples) and the number with equilibrated populations in their CSF and little to no pleocytosis (Fig. 2A; equilibrated (−)). In contrast, we estimated the percentage of samples with evidence of viral involvement in the CNS from the number with equilibrated populations in their CSF and pleocytosis (Fig. 2A; equilibrated (+)) and the number with compartmentalized populations in their CSF (Fig. 2A; compartmentalized). In these analyses we interpret equilibrated populations with pleocytosis/inflammation as an indirect marker of local viral replication, i.e. the inflammatory response to local viral replication. Based on these analyses we determined that the vast majority of samples collected during the three time periods (0–4, 5–12 and 12–24 months p.i.) had no evidence of CNS involvement (82, 78 and 90%, respectively), while the remaining samples (18, 22 and 10%, respectively) had evidence of CNS involvement. However, if viral replication and its associated markers (e.g. pleocytosis) are transient or produce small signals, these values will underestimate the actual proportion of subjects with viral replication in their CNS.

**Fig 3:**
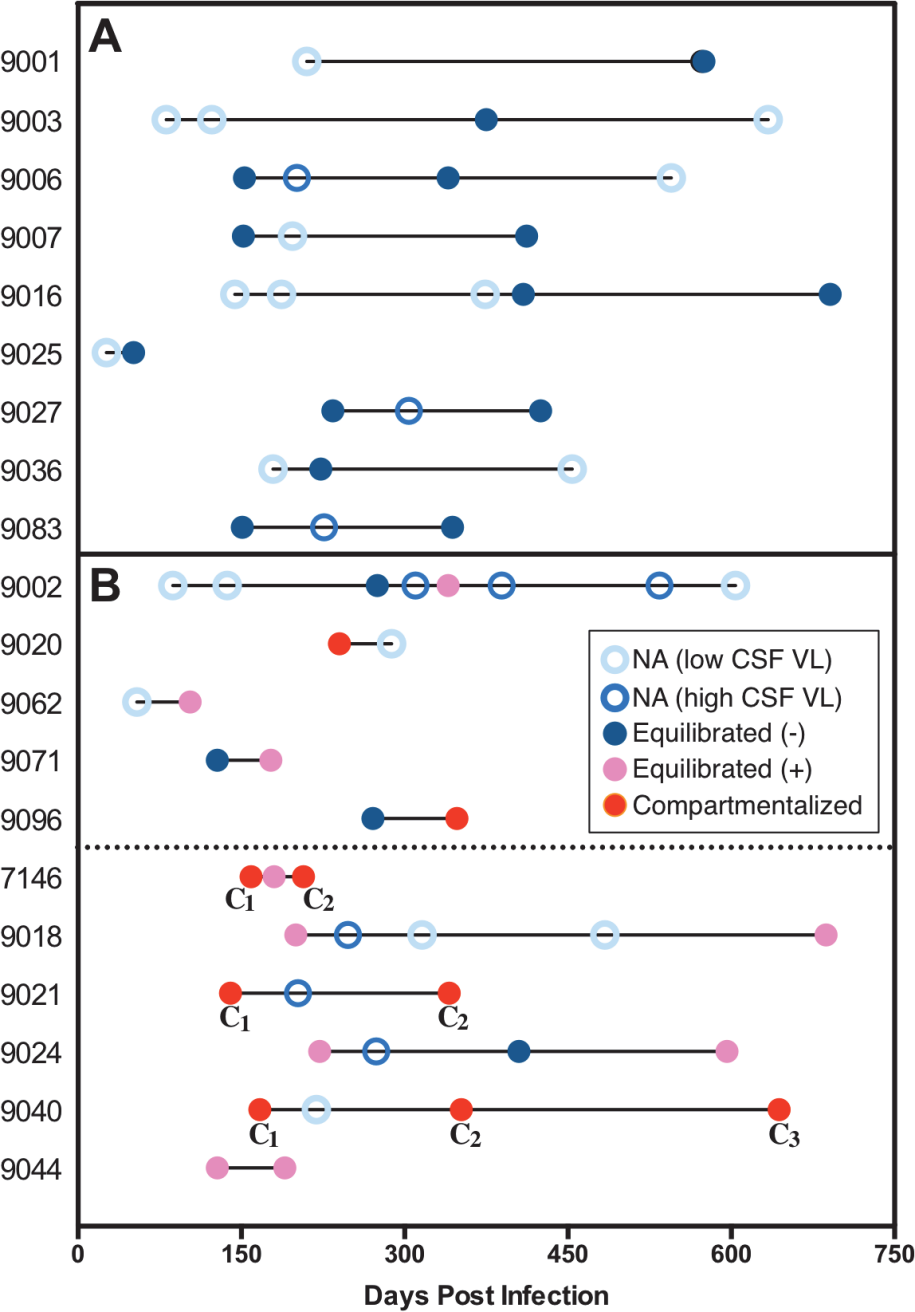
An assessment of the viral populations in the two compartments longitudinally. Examination of longitudinal relationships early during infection for 20 subjects with multiple time points where at least one time point was analyzed for viral populations. This group did not differ significantly from the 17 subjects with longitudinal samples available but not analyzed (due to low CSF viral load) in the average number of samples per person or the time span covered. Within individual subjects, the days post infection for each sample is indicated using circles, which represent the following: open light blue circle, sample not analyzed due to CSF viral load (VL) < 1,000 copies/ml (NA (low CSF VL)); open royal blue circle; sample not analyzed but CSF VL > 1,000 copies/ml (NA (high CSF VL)); closed dark blue circle, equilibrated with minimal CSF pleocytosis (CSF WBC <10 cells/μl) (Equilibrated (−)); closed pink circle, equilibrated with marked CSF pleocytosis (CSF WBC ≥10 cells/μl) (Equilibrated (+)); and closed red circle, compartmentalized. (A) Subjects with all analyzed time points equilibrated with minimal to no pleocytosis and remaining time points not analyzed. (B) Subjects with pleocytosis or compartmentalization in one time point (above dotted line) and subjects with pleocytosis, or compartmentalization in at least two time points (below dotted line). In every subject the compartmentalized (C) viral sequence population changed between sampling time points, indicated by different subscript numbers.

We used a longitudinal analysis to evaluate the persistence of viral replication in the CNS. Of the 37 subjects with longitudinal sampling, the majority (17 of 37) had low CSF viral loads at all sampling time points and were not analyzed by SGA (S1 Table). If we focus on the 20 subjects that had higher CSF viral loads (>1,000 copies of HIV-1 RNA/ml) and were analyzed by SGA, we observe that 9 (45%) had equilibrated populations with minimal to no pleocytosis at all time points analyzed (Fig. 3A). The remaining 11 subjects (55% or 30% of all longitudinally sampled subjects) all showed evidence of pleocytosis and/or compartmentalized viral replication at one or more time points within the initial two-year period (Fig. 3B) and 6 (30% or 16% of all longitudinally sampled subjects) had one of these states at two or more time points (Fig. 3B, subjects below dotted line). Thus, 55% of longitudinally sampled subjects analyzed by SGA showed evidence of viral replication and/or marked pleocytosis in the CNS at one or more time points, and 30% showed evidence of persistent viral replication in the CNS. Finally, if the initial sample showed evidence of compartmentalization or an equilibrated population with pleocytosis then subsequent samples were significantly more likely to also be compartmentalized or have pleocytosis than if the initial sample was not in one of these states (*P* = 0.001).

### Genetic evidence for the persistence of HIV-1 CNS replication

In two subjects, 9040 and 9021, a compartmentalized CNS population was observed at enrollment and for all analyzed longitudinal time points, spanning a period of 753 and 201 days p.i., respectively (one time point beyond 2 years p.i. was analyzed for subject 9040 but was not included in any overall population analysis). Further analysis of these subjects identified distinct trends of how HIV-1 becomes established in the CNS.

**Fig 4:**
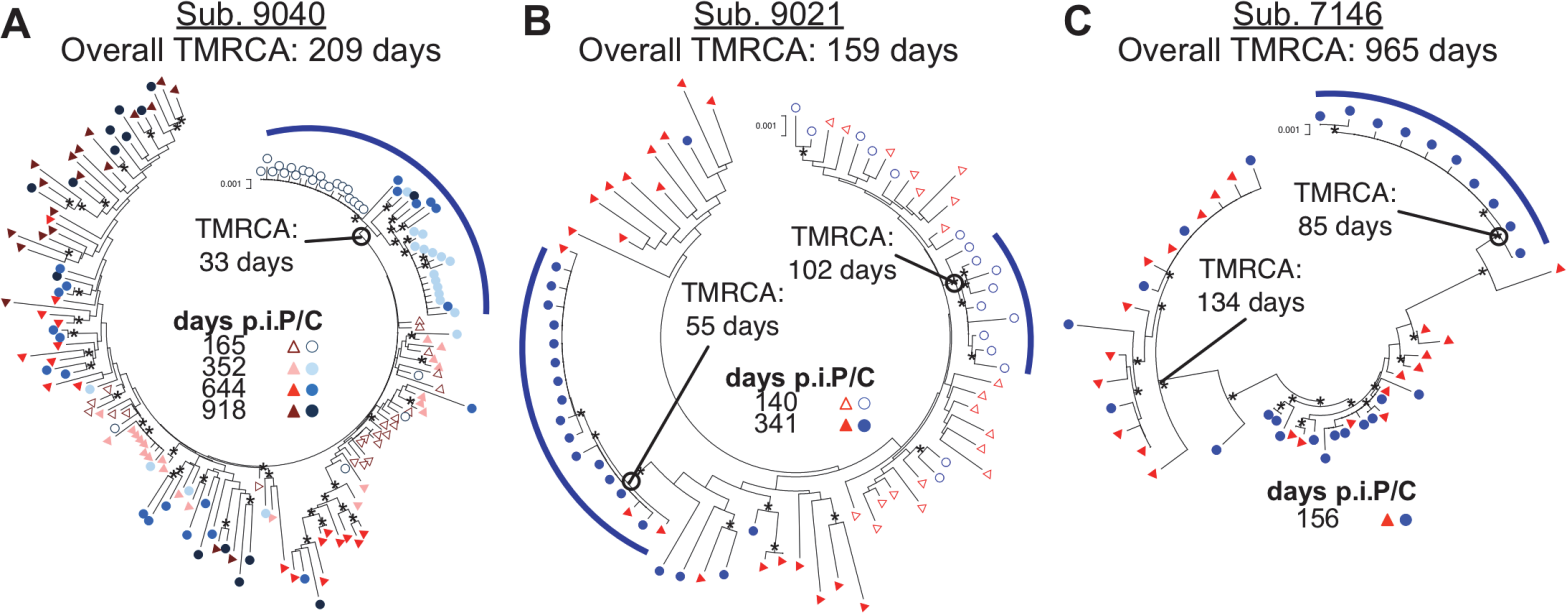
Compartmentalization can persist and evolve independently within the CSF over time. Neighbor-joining phylogenetic trees showing how compartmentalization can: (A) be persistent with multiple clonal expansions allowing recombination; (B) consist of sequential transient clonal expansions; and (C) be established with a transmitted variant. *env* sequences from the CSF are labeled with circles (C, colors designated in figure) and *env* sequences from the blood plasma are labeled with triangles (P, colors designated in figure). Days p.i. are noted. Bootstrap values ≥ 50 are indicated (*) at the appropriate nodes to highlight the more significant branch points. Genetic distance is indicated at the top of each phylogenetic tree (0.001, number of nucleotide substitutions per site between *env* sequences.) Compartmentalized populations are indicated at the appropriate node by an open circle and emphasized using a blue bar. BEAST-generated TMRCAs of the entire viral population are noted adjacent to the subject ID, and the TMRCAs of the different compartmentalized linages (subject 9040 and 9021) and transmitted parental lineages (subject 7146) are also noted.

One pattern was seen in the viral evolution in subject 9040 (Fig. 4A). In this case a compartmentalized, clonally amplified population present at the initial time point (day 165) was replaced with a second compartmentalized, clonally amplified variant at day 352, but the sampling included a recombinant between the first and second clonally amplified variants. Several recombinants between these two early populations were maintained through day 918, however, the overall population was equilibrated at this last time point. Using BEAST analysis to estimate number of generations of the viral population, the time to most recent common ancestor (TMRCA) of the blood population at the initial time point was estimated to be 209 days, reasonably consistent with the reported date of transmission (165 days prior to sampling). We also estimated that the initial clonally amplified CSF population was established 33 days prior to sampling (approximately 170 days after transmission), followed by a subsequent clonal amplification event and recombination between the two lineages. These data showed the early establishment of a lineage within the CNS that persisted over a period of at least 2 years. To our knowledge, this was the first study to show the maintenance and evolution of a compartmentalized viral population within the CNS over a long duration of time starting during early infection.

A second trend was seen for subject 9021 (Fig. 4B). Again, the reported date of transmission, 140 days prior to the first sampling date, was reasonably close to the transmission bottleneck estimated using BEAST at 159 days prior to sampling. In this subject one compartmentalized, clonally amplified lineage was detected in the CSF at the first sampling time point with an estimated age of 102 days, or starting 57 days post infection. This lineage was not present at the second sampling time point (at 341 days) but was replaced by a different compartmentalized, clonally amplified variant with an estimated age of 55 days. Thus for this subject we observed a permissive environment for viral replication in which variants were successively and independently amplified within the CNS.

### Timing of introduction of HIV-1 into the CNS

We detected compartmentalization as early as 140 days p.i. (Table 2). Using BEAST to estimate the time to most recent common ancestor (TMRCA) we were able to show that often these CSF populations were established much earlier. Four subjects with longitudinal sampling (11 samples total) had a compartmentalized CSF population detected between 5–12 months post infection (Table 2); three of these subjects had an initial clonally amplified CSF population (7146, 9021 and 9040; Fig. 4). When we determined the estimated TMRCAs of these CSF populations, we estimate the initial clonally amplified populations were all established within approximately the first four months after infection and two were established within the first two months of infection (Table 2). These data show that the CNS compartment is permissive for HIV-1 replication in at least a subset of subjects from very early times after infection.

We have recently shown that after vertical transmission to children, CNS compartmentalization can be established early via the sequestration of one of multiple transmitted variants in the CNS [14]. When we reanalyzed data from a previously described subject (7146, Fig. 4C) [4] using BEAST, we showed that the phenomenon of transmission of two variants with one sequestered in the CSF/CNS shortly after transmission also occurs in adults. In this case the two transmitted variants diverged from each other in the donor (BEAST-estimated TMRCA of 965 days but with a reported transmission date of 156 days prior to sampling) while the transmitted variants diversified in the recipient. One lineage that was present in both the blood and CSF went through a bottleneck (presumably the transmission bottleneck) at 134 days prior to sampling, again consistent with the reported transmission date of 156 days prior to sampling. The other variant was sequestered in the CNS with an estimated bottleneck of 85 days, appearing early as a clonally amplified variant. In addition, a series of recombinants between these two lineages appeared in both the blood and CSF over time. This additional mechanism for establishing a compartmentalized viral population within the CNS early following transmission was also observed for subject 9018 (Table 2).

### All transmitted variants are R5 T cell-tropic and are predominantly selected to use high levels of CD4 for entry

Macrophage-tropic HIV-1 variants can infect cells expressing low levels of CD4 while R5 T cell-tropic viruses are selected for replication in cells with high levels of CD4 for entry [24–31]. Macrophage-tropic HIV-1 is seen most reliably as a compartmentalized CSF/CNS population in a subset of individuals with HAD. To further our understanding of viral characteristics in the CNS early during infection, we analyzed the entry phenotype of viruses isolated from our adult primary infection cohort. Affinofile cells, on which CD4 and CCR5 surface expression can be differentially induced [32], are a more reproducible model for entry tropism analysis compared to primary cells [33]. We assessed entry phenotypes by measuring the ability of pseudotyped reporter viruses to enter Affinofile cells expressing either high or low levels of CD4.

**Fig 5:**
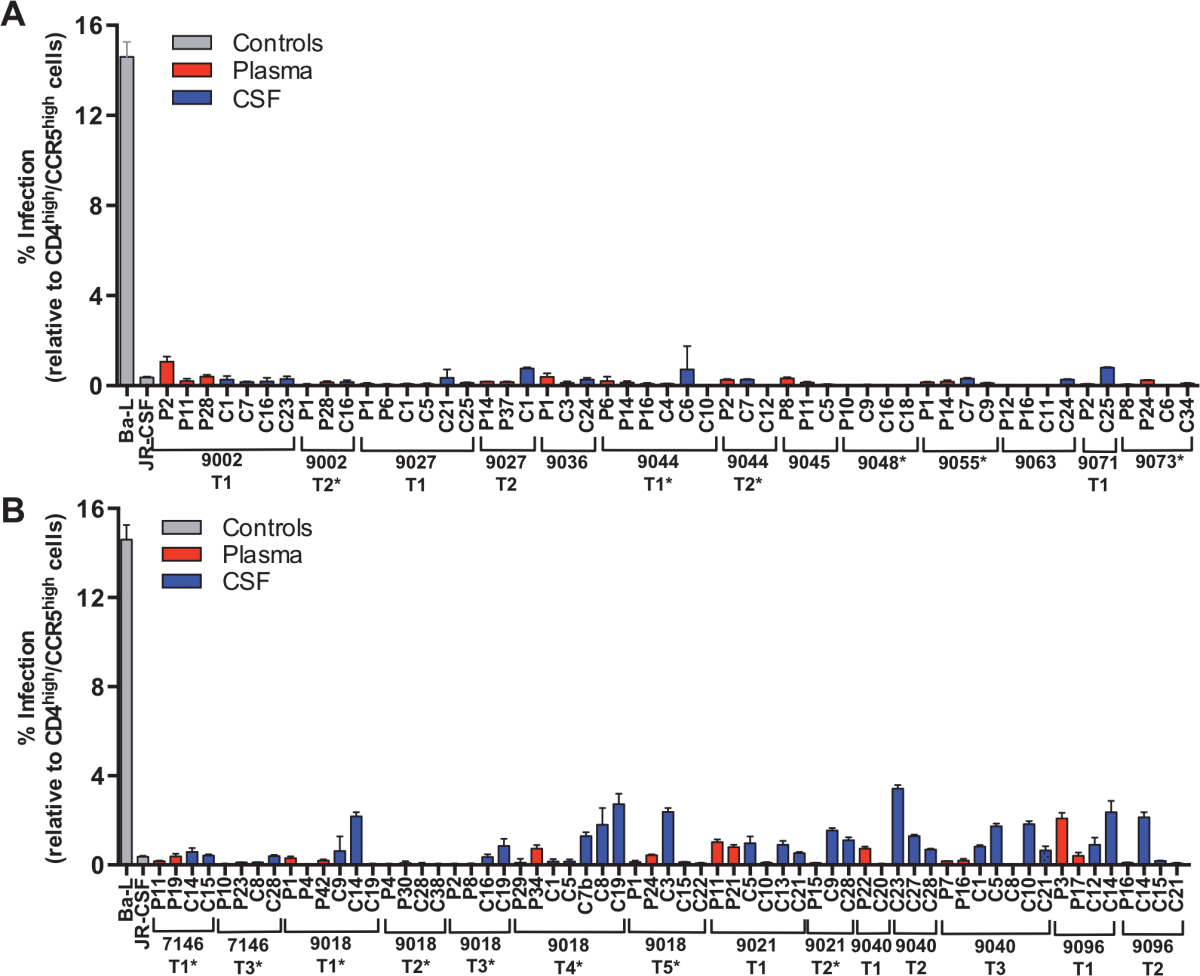
All viruses required high levels of CD4 for efficient entry, indicative of primarily being selected for replication in CD4+ T cells. Single-cycle infection of HIV-1 Env-pseudotyped reporter viruses on CD4^low^CCR5^high^ 293-Affinofile cells [28]. Receptor expression was measured as follows: CD4^low^ = 972 receptors/cell, CD4^high^ = 72,041 receptors/cell, CCR5^high^ = 15,636 receptors/cell. The data are averaged from triplicate wells for each of 2 to 3 *env* clones that were generated per indicated amplicon. Amplicons were selected for cloning to represent different portions of the phylogenetic tree. Subjects with no evidence of CNS compartmentalization (i.e. equilibrated) are shown in panel (A); and subjects with CNS compartmentalization at one or more time points are in panel (B). Longitudinal time points are indicated (T1, T2, T3, etc.) and samples with marked pleocytosis are noted (*).

Viruses pseudotyped with Env proteins derived from 24 subjects, representing all phylogenetic states and a wide range of days p.i., all required high levels of CCR5 and CD4 for efficient entry and were considered R5 T cell-tropic (Fig. 5A-B). However, Env proteins from CSF samples containing compartmentalized viral lineages (all collected more than 4 months post infection) were significantly better at entering cells expressing low CD4 than Env proteins derived from equilibrated CSF samples (ANOVA; P<<0.001). While this enhanced ability to enter low CD4 cells did not near the level of a macrophage-tropic Env protein (Fig. 5A-B, Ba-L), the detectably elevated levels suggest that compartmentalized lineages may be adapting to enter low CD4 cells in the CNS (discussed below). In contrast, viruses isolated from the CSF of adults within four months of infection are uniformly poor at infecting low CD4 cells, indicating that they have been selected for replication in T cells. This is consistent with multiple studies showing that macrophage-tropic viruses are not transmitted [16,27,28,34–39]. Together these results indicate that the CNS is initially exposed to R5 T cell-tropic variants and that viruses in the CNS remain R5 T cell-tropic for the first two years of infection, but the data provide suggestive evidence that the evolution of macrophage tropism may begin during this period.

We also assessed the infectivity of a subset of the pseudotyped viruses on monocyte-derived macrophages (MDMs) generated from three separate donors. Infectivity was first assessed on Affinofile cells expressing high levels of CD4, with equal levels of infectious virus then added to each of the MDM preparations. There was general concordance between infectivity on Affinofile cells and infectivity on MDMs, although the level of infectivity differed significantly between the three donors (S2 Fig.). Viruses representing subjects with equilibrated populations and with low infectivity on Affinofile cells with low CD4 (Fig. 5A) had low infectivity on MDM (from subjects 9027, 9045, 9055, 9063, and 9073; S2 Fig.).

Viruses from four of five subjects with CNS compartmentalization were also assessed for their ability to enter Affinofile cells expressing low levels of CD4 and MDMs. Both blood- and CSF-derived viruses from one subject (subject 9096) were observed to have intermediate infectivity on low CD4 Affinofile cells (Fig. 5B) and MDMs (S2 Fig.). Similarly, two of the compartmentalized subjects (9018 and 9021) had CSF-derived viruses with elevated infectivity of low CD4 Affinofile cells (Fig. 5B) and MDMs (S2 Fig.). Finally, both blood- and CSF-derived viruses from one compartmentalized subject (subject 7146) were unable to efficiently enter both low CD4 expressing Affinofile cells and MDMs (S2 Fig.). Thus infectivity of MDMs is generally consistent with the conclusions derived from infection of Affinofile cells although the variability of infectivity of MDMs between donors precludes an accurate assessment of the range of CD4 entry phenotypes that can be observed using Affinofile cells.

## Discussion

Independent HIV-1 replication in the CNS has been associated with neurological disorders [10,40,41] and may represent a distinct reservoir from that found in the blood and lymphoid tissue [42,43]. We examined the virologic characteristics associated with early CNS infection through analysis of paired cross-sectional and longitudinal blood plasma and CSF samples from a large cohort of 72 ART-naïve subjects infected with HIV-1 for less than two years. Our current study significantly builds upon a previous preliminary study by our group [4], enabling us to propose a model with four distinct states to describe the relationship between viral populations in the CSF/CNS and viral populations within the peripheral blood during early HIV-1 infection. These states are based upon details revealed by the current study on mechanisms of establishment of viral compartmentalization within the CNS, relationships between cellular inflammation, HIV-1 RNA levels and phylogenetic state, and insight into longitudinal maintenance and evolution of compartmentalization.

**Fig 6:**
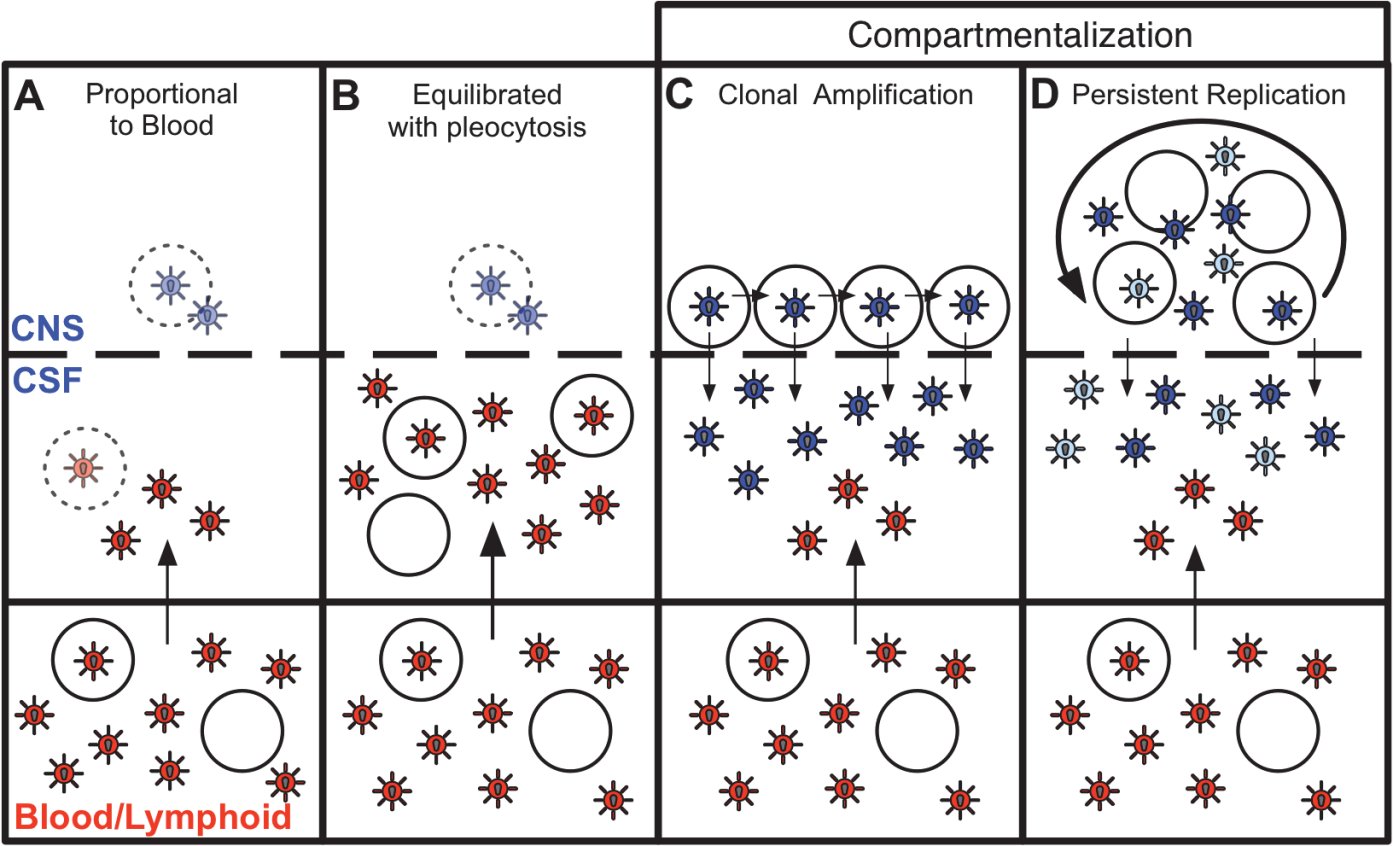
Four states can define the relationship between virus in the CSF/CNS and blood early during infection. Blood and CSF/CNS compartments are indicated. Blood plasma viral variants are represented by the red virus particles; and CNS viral variants are represented by the blue virus particles. CD4+ T cells are represented by open circles. Arrows indicate direction of virus movement between compartments. (A) State with a CSF HIV-1 RNA level 1–2% of the viral load in the blood and defined by minimal to no local CNS replication or inflammation, resulting in an equilibrated state between the two compartments (when CSF viral load high enough for analysis). Transparent infected CD4+ T cell represents potential local CNS replication that may be obscured by the import of virus into the CNS from the periphery. (B) State of equilibration between CSF and blood accompanied by high levels of pleocytosis, potentially caused by local CNS replication. Transparent infected CD4+ T cell represents potential local CNS replication likely obscured by the high levels of virus secreted by the infiltrating CD4+ T cells. (C) State of CSF/CNS clonal amplification of identical or nearly identical variants within CD4+ T cells. These clonally amplified populations are characterized by low diversity, signified by all CSF viruses in a single shade of blue. (D) State of genetically complex, compartmentalized viral replication within CD4+ T cells in the CSF/CNS indicative of persistent replication beyond a single clonal amplification event; this complexity is signified by CNS viral variants in multiple shades of blue.

The first state (Fig. 6A) was observed in subjects with little evidence of CNS replication or pleocytosis, with CSF HIV-1 RNA concentrations proportionally 1–2% of the viral load in the periphery (Fig. 2B). In many of these subjects, the CSF HIV-1 RNA level was very low, below the limit of detection of standard assays. Minimal CSF viral burden has been observed in a prior report on a portion of this primary infection cohort [5]. With little or no pleocytosis, HIV-1 is likely entering the CSF/CNS at low levels via incomplete partitioning of virus at the blood-brain barrier, or low level trafficking of immune cells, including small numbers of infected CD4+ T cells. In this circumstance the viral population is very similar to the population in the blood. It is possible that some HIV-1 is replicating independently in the CNS at low levels in these subjects, but we were not able to detect these putative genetic variants above the low level background of virus recently imported from the periphery into the CSF/CNS. An argument in favor of even this low level viremia in the CSF being the result of T cell trafficking is the observation that in neuro-asymptomatic subjects with CD4+ T cells below 50 cells/ul in the blood the viral load in the CSF is on average lower than in subjects with higher CD4+ T cell counts [44,45].

In a second state (Fig. 6B), we observed a relationship between equilibrated viral populations with elevated viral load and high levels of pleocytosis (Fig. 2C). These equilibrated populations were most likely the result of the release of virus from increased numbers of infected CD4+ T cells trafficking from the periphery into the CNS. Though pleocytosis of > 10 cells/ul might have been due to a variety of inflammatory conditions (e.g. neurosyphilis as documented in one individual), it is most likely in response to HIV-1 replication in these PHI subjects. We screened for syphilis in this cohort, and detailed clinical and imaging assessment did not reveal other contributing causes of pleocytosis. Furthermore, other ‘background’ non-HIV causes of CSF WBC ≥ 10 cells/ul are unlikely in these subjects, as our parallel studies of 54 HIV-uninfected volunteers recruited from the similar local community demonstrated median CSF WBC counts of 1 cell/ul (IQR 0–2), and none of these 54 subjects had a CSF WBC as high as 10 [5]. In the setting of pleocytosis, while low levels of local CNS replication may have been occurring, the virus imported from the periphery by infected CD4+ T cells dominated the population as it raised the CSF HIV-1 RNA concentration by release of virus imported from the blood. If the inflammatory immune response was successful, pleocytosis might eventually result in low CSF viral loads, a condition observed in a small subset of subjects with pleocytosis but very low levels of virus in the CSF (S1 Table). Pleocytosis was also observed in several subjects with an intermediate viral population phenotype and half of the subjects with compartmentalized viral populations (Fig. 2D), suggesting pleocytosis may result in dynamic changes in the viral population in the CSF. An association between equilibrated compartments and high pleocytosis was also observed in a previous study analyzing four HIV-infected subjects during therapy interruption [46].

In a third state (Fig. 6C), we observed clonally amplified CSF populations of low complexity (Fig. 1 and 4) representing the recent expansion of identical or nearly identical variants that required high levels of CD4 for entry (R5 T cell-tropic; Fig. 5). High levels of pleocytosis were observed in approximately half of the subjects with clonally amplified CSF populations, making it possible that the influx of activated CD4+ T cells may also have provided cellular targets for further transient amplification of a CSF variant. We [47] (Dukhovlinova et al., in preparation) and others [48–50] have observed clonal amplification in the genital tract as well as in the CSF both early [4,14] and at later times in infection [41]. Clonal amplification appears to be a distinct type of virus-host interaction where infection of a population of CD4+ T cells in a compartment is a low probability event and when it occurs there is transient rapid expansion of the viral population. Due to the daily rapid turnover of the CSF viral RNA load, the elevated CSF viral RNA load that is often observed during clonal amplification, and the fact that these clonally amplified lineages generate their own diversity that can persist within the CNS, it is highly unlikely that clonally amplified virus represents virus produced from a single cell. The detection of clonally amplified populations in the CSF within the first year of infection has allowed us to estimate the establishment of these populations within the CNS to within the first 2–6 months (Table 2, Fig. 4), and such amplified populations were detected in 8% of subjects in this study within the first year.

In the final state, we observed more genetically complex compartmentalized viral replication within the CSF/CNS (Fig. 6D) indicative of persistent replication beyond a single clonal amplification event. In an effort to get a more complete view of the interaction of the virus within the CNS at these early times of infection we have interpreted the presence of persistent replication in the CNS based on four criteria: i) sequential clonal amplification events that indicated a permissive CNS environment for viral replication (Fig. 4B); ii) overlapping clonal amplification events that gave rise to compartmentalized recombinants showing continuous replication between the sampled time points (Fig. 4A); iii) intermittent compartmentalization and pleocytosis suggesting an inflammatory immune response to ongoing replication (Fig. 3B); and iv) sequestration of a transmitted variant within the CNS (Fig. 4C). Collectively these markers defined approximately 30% of subjects in the first two years as having evidence of viral replication in the CNS in at least one time point, and 16% having evidence of replication and/or inflammation at multiple time points within this period. This suggests that the CNS compartment is permissive for HIV-1 replication in at least a subset of subjects from a very early period after infection.

Entry tropism analysis revealed that all compartmentalized variants required high levels of CD4 for entry. It is now widely described in the literature that macrophage-tropic variants utilize low levels of CD4 for entry [24–31], are not transmitted, and that the transmitted virus is R5 T cell-tropic [16,27,28,34–39], an understanding further supported by our phenotypic analysis (Fig. 5). Our finding that the viruses involved in this early persistent CNS replication were adapted to replication in CD4+ T cells is distinct from previous studies of individuals with HAD where genetically complex compartmentalized CSF populations that had been replicating as an isolated population had evolved to replicate in macrophages/microglia [41]. Thus, adaptation to use low levels of CD4 for entry, a hallmark of macrophage tropism, is not a feature of the transmitted virus and does not evolve during the early stages of CNS infection in adults, at least as reflected in the compartmentalized virus detected in the CSF. However, we do note that the compartmentalized viruses from the CSF show a small but statistically significant increase in the ability to enter cells with low levels of CD4 compared to CSF virus from equilibrated subjects (Fig. 5B). One explanation for this small difference is that the virus in the CNS is carrying out at least a portion of its replication in a cell with low levels of CD4 which allows for at least a low level of selection for a low CD4 entry phenotype. We found no consistent differences in glycosylation site count or positions, or consistent sequence changes in sites previously described as being associated with macrophage tropism in comparing the viral sequences from the plasma to the compartmentalized sequences in the CSF (S2 Table and S3).

The only CNS tissue that is readily sampled in volunteer human subjects is CSF, which, though not identical to brain, is produced within the brain in the choroid plexus, and reflects brain inflammation and infection in the context of CNS infections including HIV-1. Measures of immune activation, HIV-1 burden, and neural injury detected in CSF are markers of brain involvement in HIV-1 that correlate to clinical and pathologic disease [51]. While the cellular source of HIV-1 RNA detected in the CSF is not certain and may differ during different stages of infection [52], compartmentalization of HIV-1 detected in CSF associates with clinical dementia in humans [10] and immunopathology in the brain in rhesus macaques [53]. Despite limitations of generalizing CSF findings to those of the CNS more broadly, our studies have used the best methods available in living humans to assess HIV-1 populations derived from the CNS in a unique cohort of subjects enrolled during primary HIV-1 infection. Our results show that in cross-sectional analysis over the first two years of HIV-1 infection, 30% of subjects have evidence of either local viral replication in the CNS or a robust CNS inflammatory response, and that in approximately 16% of subjects this CNS involvement can persist over time. We have found that the viral population in the CSF is dynamic as the result of local replication and/or the influx of virus in infected CD4+ T cells as part of an inflammatory response. This early viral replication in a subset of subjects may represent an inability to protect the CNS from infection, potentially leading to HAND later in infection, and may also define a distinct reservoir of infected cells within the body. Longitudinal follow-up of these subjects to examine the long-term impact of the presence of early active HIV-1 replication in the CNS will help to define the significance of these findings for clinical neurologic disease outcomes and compartmentalized viral reservoirs in the setting of HIV-1.

## Materials and Methods

### Ethics statement

The study was approved by the Institutional Review Boards at UCSF, Yale University, and the University of North Carolina at Chapel Hill. All study participants were adults (age ≥ 18 years). Written informed consent was obtained from all participants.

### Study design

We assessed samples obtained through an observational longitudinal neurological study of primary HIV-1 infection to determine viral characteristics associated with early HIV-1 CNS infection. Subjects were referred from the community and were eligible if they met prospectively determined criteria for laboratory confirmation of primary HIV-1 infection, as previously described [4]. Subjects were screened at study enrollment for systemic syphilis by blood RPR testing. Subsequent blood and CSF samples were tested for RPR and VDRL, respectively, if an outside test suggested syphilis exposure or CSF WBC was markedly elevated compared to that in an earlier longitudinal sample. No subjects had clinical evidence of other inflammatory neurologic disorders such as multiple sclerosis or CNS opportunistic infections based on interview and examination by an HIV-1 neurologist at each visit. A total of 57 of the 72 subjects volunteered to participate in magnetic resonance imaging of the brain for the overall study protocol; scans were reviewed by a neuroradiologist and none revealed evidence of encephalitis, tumor or opportunistic infection. Blood and CSF samples were collected at enrollment, six weeks, and every six months thereafter. This analysis included samples obtained up to two years post-infection from subjects enrolled prior to 4/1/2012. CSF and plasma HIV-1 RNA concentrations were determined as described [4]; paired samples were selected for further SGA analysis if CSF HIV-1 RNA was greater than1,000 copies/ml (to ensure adequate sampling). Samples with lower viral loads could be analyzed if larger volumes were committed to concentrate the virus, but we chose to use a cut-off of 1,000 viral RNA copies/ml for these studies. Primary study endpoints included SGA of the HIV-1 *env* gene for viral genetic compartmentalization and phenotypic analyses, CSF and blood HIV-1 RNA concentrations, measures of CSF cellular inflammatory response (white blood count, WBC) and blood brain barrier disruption (CSF:plasma albumin ratio).

### Single genome amplification

When collected, CSF samples were initially placed on wet ice and delivered to the lab for processing within one hour. For virology analyses, CSF samples were centrifuged at 1200 x g for 10 min to remove contaminating cells or cellular debris, and the supernatant was subsequently aliquoted and stored at −70°C for later assay. Viral RNA was isolated as previously described from the CSF supernatant [4]. Briefly, RNA was isolated from samples (140 μl) with viral loads >10,000 copies/ml using the QIAmp Viral RNA Mini kit (Qiagen). To increase template number, samples with viral loads <10,000 copies/ml were first pelleted by ultracentrifugation. cDNA was generated using an oligo-d(T) primer. Single genome amplification/template endpoint dilution PCR [38] of the *env* gene through the 3’ LTR U3 end was conducted using the cDNA as template as previously described [4,14]. Sequences for full-length *env* were generated (samples analyzed previously were sequenced from the start of V1 through the ectodomain of gp41 [4]).

### Phylogenetic analysis of *env* viral sequences

Phylogenetic analysis were conducted in a manner similar to our previous study [14]. In brief, DNA sequences were aligned (MUSCLE) [54–56] using EBI web tools [57], and phylogenetic trees were generated (neighbor-joining method, MEGA 4.0 [58]). Phylogenetic states were determined by statistical evaluation using the Slatkin-Maddison (SM) test [18] as previously described [14], Wright’s measure of population subdivision (F_st_) [19,20] and the Nearest-neighbor statistic (S_nn_) [21]. CSF populations were defined as being compartmentalization if all three statistical tests (SM, F_st_ and S_nn_) yielded significant results *(P* values < 0.05) or equilibrated if one or more of tests yielded non-significant results. Low bootstrap values were used (≥35) because of the overall low diversity of the viral populations early after infection. Clonally amplified lineages (short branch lengths with bootstrap values ≥ 99 and a clade of ≥ 3 variants) were also identified. No contamination occurred between samples (S3 Fig.). Sequences for subjects 9002 (338 d.p.i.), 9007, 9018 (200 d.p.i.), 9025, 9037, 9039, 9040 (165 d.p.i.), and 7146 were generated in our previous study [4].

### Bayesian analysis

A Bayesian Markov Chain Monte Carlo (MCMC) approach using BEAST v.1.6.1 [15] estimated the TMRCA for each viral population. A substitution rate of 1.5x10^−5^ substitutions/site/generation and standard deviation of 3.0x10^−6^ were fixed under a lognormal relaxed clock (uncorrelated) model. The rate was calculated via tip dating, using a consensus sequence (set as day 0), and the estimated days post infection. The HKY nucleotide substitution model had estimated base frequencies and a gamma-distributed rate heterogeneity (4 gamma categories). A coalescent Bayesian Skyline tree prior with a Piecewise-constant skyline model was used (10 groups). The MCMC algorithm was run for 30 million generations, logging every 1000 and with a 10% burn-in. The results from at least two independent runs were combined, and the effective sample size for all estimates was >200. A generation time of 1.0 day was used.

### Construction of HIV-1 *env* clones

Full length HIV-1 *env* genes were re-amplified from the first-round SGA products as previously described [41]. The PCR product was cloned into the pcDNA3.1D/V5-His-TOPO expression vector (Invitrogen) using the pcDNA 3.1 directional TOPO expression kit (Invitrogen).

### Cells

293T cells were cultured in Dulbecco’s modified Eagle medium (DMEM) supplemented with 10% fetal bovine serum (FBS) and 100 mg/ml of penicillin and streptomycin. 293-Affinofile cells [32], generously provided by Dr. Ben-Hur Lee, were maintained in DMEM supplemented with 10% dialyzed FBS (12–14 kD dialyzed; Atlanta Biologicals) and 50 mg/ml blasticidin (D10F/B).

### Env-pseudotyped viruses

Env-pseudotyped luciferase reporter viruses were generated as previously described [14]. Briefly, 293T cells were cotransfected with an *env* expression vector and the pNL4-3.LucR-E- HIV-1 backbone (obtained from the NIH AIDS Research and Reference Reagent Program, Division of AIDS, NIAID, NIH) using the Fugene 6 transfection reagent and protocol (Roche). Transfection medium was replaced with fresh culture medium five hours post-transfection and the cells were incubated at 37°C for 48 hours, after which viral supernatants were filtered with 0.45 μM filters (Millipore) and stored at −80°C.

### 293-Affinofile cellular surface expression of CD4 and CCR5

293-Affinofile cell [32] CD4 and CCR5 receptor expression was induced with doxycycline (doxy; Invitrogen) and ponasterone A (ponA; Invitrogen), respectively, as previously described [14]. Briefly, cells were induced at two conditions: CD4^high^/CCR5^high^ (6 ng/ml doxy and 5 μM ponA, respectively) and CD4^low^/CCR5^high^ (0 ng/ml doxy and 5 μM ponA). CD4 and CCR5 receptor expression was measured using quantitative fluorescence-activated cytometry (qFACS) following staining with either phycoerythin (PE)-conjugated anti-human CD4 antibody (clone Q4120, BD Biosciences) or PE-conjugated mouse anti-human CCR5 antibody (clone 2D7, BD Biosciences), and surface levels were calculated using *QuantiBRITE* beads (BD Biosciences).

### Single-cycle infection of 293-Affinofile cells

As described previously [14], Env-pseudotyped luciferase reporter viruses were first titered on 293-Affinofile cells [32] expressing CD4^high^/CCR5^high^. For viral infections, black tissue culture plates (96 wells) were coated with 10% poly-L-lysine and seeded with 293-Affinofile cells (1.85 x 10^4^ cells/well). Eighteen to 24 hours later, expression of CD4 and CCR5 was induced at CD4^high^/CCR5^high^ and CD4^low^/CCR5^high^. Eighteen to 24 hours later, the induction medium was removed and replaced with 100 μl of fresh, warmed culture medium containing Env-pseudotyped virus. The plates were spinoculated [59] at 2,000 rpm for 2 hours at 37°C, and incubated for 48 hours at 37°C. Infection medium was removed, cells were lysed, and luciferase activity was assayed using the luciferase assay system (Promega). Clone sequences were not compared to the original parental sequence prior to pseudotyping and Affinofile cell infection. Instead, entry tropism data for each parental amplicon included three replicates from 2–3 clones derived from the same parental amplicon. In this analysis we assume any PCR-introduced error would either not change the entry phenotype or would create a nonfunctional protein which would not be included in the subsequent analysis. The concordance of the entry phenotype of the replicate clones was taken to represent the phenotype of the amplicon sequence.

### Statistical analysis

Compartmentalization was assessed statistically using the Slatkin-Maddison test for gene flow [18], Wright’s measure of population subdivision (F_st_) [19,20] and the Nearest-neighbor statistic (S_nn_) [21]. Differences between groups were examined for statistical significance using the Mann-Whitney Test. The one exception was our analysis examining whether *env* genes derived from samples with and without CSF compartmentalized lineages differed in their ability to enter Affinofile cells expressing low levels of CD4 expression. We used a linear model to perform this analysis (performed in [R]) and used the stepAIC function to perform stepwise model selection. All correlations employed Spearman’s rank correlation coefficient. For all statistical tests, *P* values less than 0.05 were considered significant.

### Nucleotide sequence accession numbers

The HIV-1 *env* nucleotide sequences determined in this study have been deposited in GenBank under accession numbers KM353586-KM355197

## Supporting Information

S1 Fig Samples in each phylogenetic group without pleocytosis. Pie chart showing the percent of samples in each phylogenetic state exhibiting minimal to no CSF pleocytosis (CSF WBC <10 cells/μl). States represented include: Not Analyzed by SGA, due to CSF viral load <1,000 copies/ml; Equilibrated (−), CSF WBC <10 cells/μl; and Compartmentalized.
(TIFF)

S2 Fig Pseudotyped reporter virus infectivity of MDMs isolated from three donors. MDMs were infected with two positive controls that are known to be macophage-tropic (4051_C3 [41] and Ba-L [60]), two negative controls that are known to be T cell-tropic (4051_P8 [41] and JRCSF [61]) and pairs of pseudoviruses from nine subjects described in this study. Each pair was comprised of a CSF-derived virus (“C” clone) and a plasma-derived virus (“P” clone). Each virus was used to infect three replicate wells of cells from donors 1 and 3 and two replicate wells from donor 2. MDMs were generated and infected using the protocol described in Joseph et al. [62]. Briefly, monocytes were isolated from the blood of three healthy donors and differentiated for seven days in medium containing recombinant human macrophage colony stimulating factor (M-CSF). MDMs were then infected with the volume of each pseudovirus stock that we previously determined to generate 800,000 relative light units (RLU) of luciferase expression when used to infect maximally induced Affinofile cells. Five days after infection, MDMs were lysed and luciferase expression was measured. The y-axis shows the mean RLUs from replicate wells.
(TIFF)

S3 Fig No contamination was observed between subjects. Neighbor-joining phylogenetic tree (radial topology). *env* sequences from the CSF are labeled with solid blue circles and *env* sequences from the blood plasma are labeled with solid red triangles. Genetic distance is indicated at the bottom of the figure (0.001) and indicates the number of nucleotide substitutions per site between *env* sequences. Each subject ID and corresponding sampling time point are indicated.
(TIFF)

S1 Table Virologic and clinical characteristics for subjects not analyzed by SGA. (DOCX)

S2 Table Env glycosylation site analysis of compartmentalized subjects. (DOCX)

S3 Table Selective analysis of amino acid differences between compartmentalized and plasma populations in subjects analyzed for entry tropism in MDMs. (DOCX)
