# Supplementary figures and images for: Compartmentalized Replication of R5 T Cell-Tropic HIV-1 in the Central Nervous System Early in the Course of Infection
Source: PLoS Pathog. 2015 Mar 26;11(3):e1004720. doi: 10.1371/journal.ppat.1004720 (PMC4374811; doi:10.1371/journal.ppat.1004720)

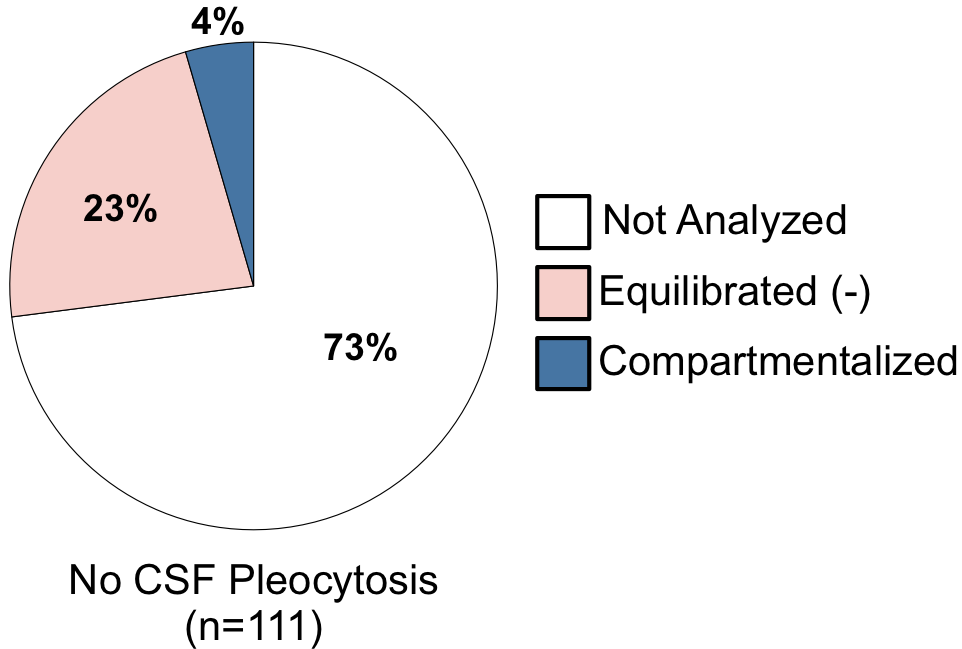

Supplement: S1 Fig — Pie chart showing the percent of samples in each phylogenetic state exhibiting minimal to no CSF pleocytosis (CSF WBC <10 cells/μl). States represented include: Not Analyzed by SGA, due to CSF viral load <1,000 copies/ml; Equilibrated (−), CSF WBC <10 cells/μl; and Compartmentalized. (TIFF) [file ppat.1004720.s001.tiff]

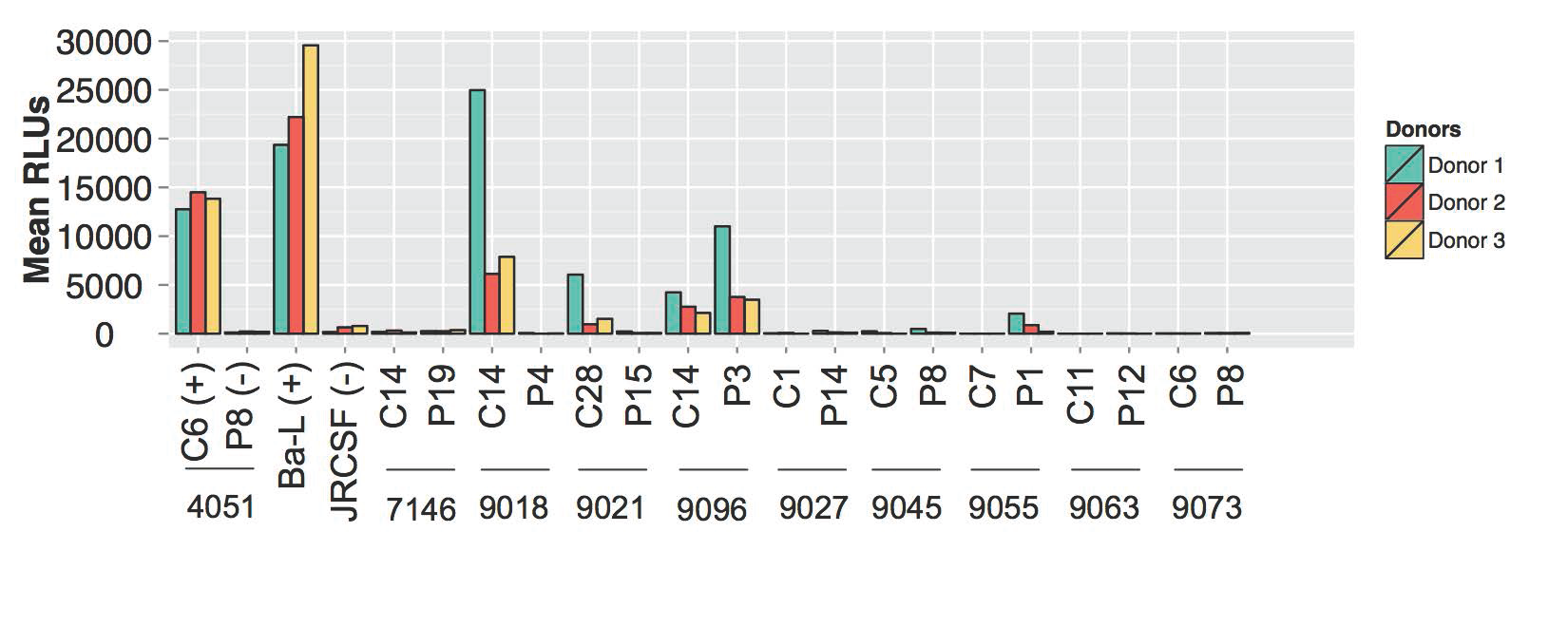

Supplement: S2 Fig — MDMs were infected with two positive controls that are known to be macophage-tropic (4051_C3 [41] and Ba-L [60]), two negative controls that are known to be T cell-tropic (4051_P8 [41] and JRCSF [61]) and pairs of pseudoviruses from nine subjects described in this study. Each pair was comprised of a CSF-derived virus (“C” clone) and a plasma-derived virus (“P” clone). Each virus was used to infect three replicate wells of cells from donors 1 and 3 and two replicate wells from donor 2. MDMs were generated and infected using the protocol described in Joseph et al. [62]. Briefly, monocytes were isolated from the blood of three healthy donors and differentiated for seven days in medium containing recombinant human macrophage colony stimulating factor (M-CSF). MDMs were then infected with the volume of each pseudovirus stock that we previously determined to generate 800,000 relative light units (RLU) of luciferase expression when used to infect maximally induced Affinofile cells. Five days after infection, MDMs were lysed and luciferase expression was measured. The y-axis shows the mean RLUs from replicate wells. (TIFF) [file ppat.1004720.s002.tiff]

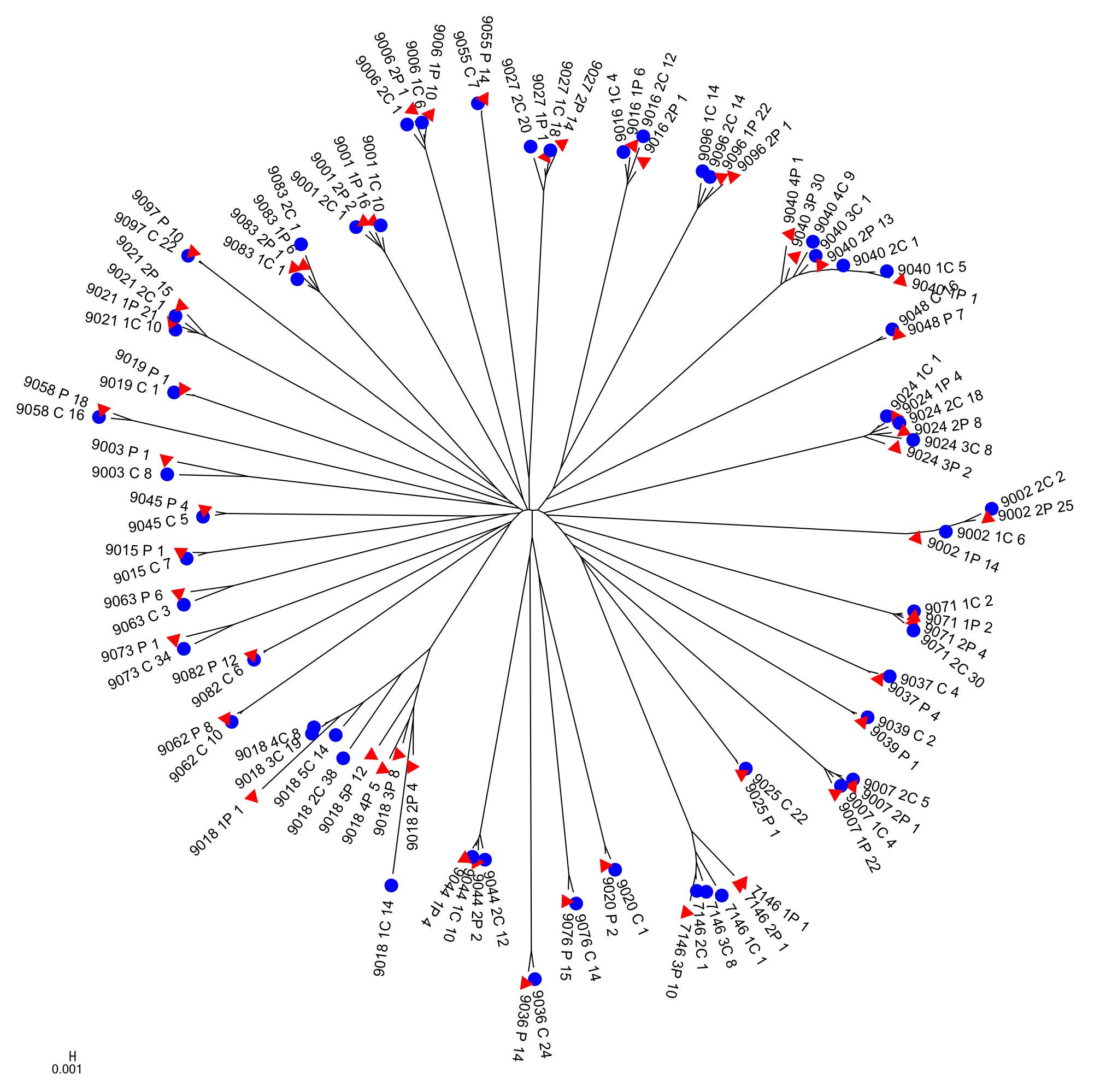

Supplement: S3 Fig — Neighbor-joining phylogenetic tree (radial topology). env sequences from the CSF are labeled with solid blue circles and env sequences from the blood plasma are labeled with solid red triangles. Genetic distance is indicated at the bottom of the figure (0.001) and indicates the number of nucleotide substitutions per site between env sequences. Each subject ID and corresponding sampling time point are indicated. (TIFF) [file ppat.1004720.s003.tiff]
