# Supplementary material for: Compartmentalized Replication of R5 T Cell-Tropic HIV-1 in the Central Nervous System Early in the Course of Infection
Source: PLoS Pathog. 2015 Mar 26;11(3):e1004720. doi: 10.1371/journal.ppat.1004720 (PMC4374811; doi:10.1371/journal.ppat.1004720)
Supplement: S1 Table — (DOCX) [file ppat.1004720.s004.docx]

**S1 Table. Virologic and clinical characteristics for subjects not analyzed by SGA.**

| Subject ID^a^ | Days p.i.^b^ | VL Plasma^c^ | VL CSF^d^ | CD4^e^ | CSF WBC^f^ | Albumin ratio^g^ |
| --- | --- | --- | --- | --- | --- | --- |
| 9004 | 123 | 3.97 | 1.69 | 587 | 2 | 4.2 |
| 9005 | 89 | 2.98 | 1.69 | 656 | 5 | 2.0 |
|  | 139 | 3.32 | 1.69 | 627 | 3 | 2.3 |
| 9008 | 148 | 2.92 | 1.69 | 758 | 1 | 4.3 |
|  | 203 | 3.89 | 1.69 | 715 | 0 | 4.7 |
| 9010 | 237 | 3.01 | 2.31 | 497 | 11 | 5.6 |
|  | 302 | 3.51 | 1.69 | 538 | 6 | 3.8 |
|  | 439 | 2.87 | 1.69 | 242 | 3 | 5.4 |
|  | 547 | 4.24 | 1.81 | 175 | 5 | 5.3 |
| 9012 | 106 | 4.87 | 2.60 | 601 | 8 | 11.1 |
|  | 162 | 5.58 | 2.89 | 627 | 7 | 11.9 |
|  | 293 | 5.03 | 2.69 | 544 | 7 | 10.4 |
|  | 475 | 4.86 | 2.47 | 544 | 8 | 10.4 |
| 9017 | 50 | 4.24 | 2.18 | 361 | 14 | 4.5 |
| 9023 | 82 | 4.94 | 2.58 | 305 | 0 | 10.1 |
| 9026 | 46 | 4.70 | 2.62 | 317 | 6 | 6.8 |
| 9029 | 376 | 4.10 | 2.95 | 410 | 1 | 6.4 |
|  | 418 | 4.02 | 2.33 | 520 | 1 | 5.5 |
| 9030 | 304 | 4.10 | 2.05 | 447 | 2 | 5.4 |
|  | 409 | 3.94 | 2.65 | 501 | 8 | 6.0 |
| 9034 | 54 | 3.89 | 1.69 | 854 | 3 | 4.5 |
|  | 98 | 3.51 | 2.08 | 787 | 3 | 4.3 |
| 9035 | 51 | 4.46 | 1.75 | 576 | 2 | 5.0 |
| 9038 | 92 | 1.69 | 1.69 | 1234 | 6 | 7.4 |
|  | 155 | 1.69 | 1.69 | 1399 | 4 | 6.7 |
|  | 289 | 1.69 | 1.69 | 1497 | 2 | 7.5 |
|  | 484 | 1.69 | 1.69 | 1251 | 6 | 7.8 |
|  | 714 | 1.69 | 1.69 | 898 | 0 | 8.0 |
| 9042 | 42 | 4.24 | 1.85 | 854 | 2 | 4.8 |
| 9043 | 102 | 3.50 | 1.69 | 1143 | 4 | 12.1 |
|  | 185 | 3.61 | 1.69 | 1076 | 6 | 10.1 |
|  | 295 | 4.08 | 2.09 | 891 | 6 | 10.9 |
| 9046 | 74 | 2.83 | 1.69 | 596 | 30 | 12.7 |
| 9047 | 91 | 4.35 | 1.56 | 586 | 1 | 7.4 |
| 9049 | 176 | 3.64 | 1.86 | 735 | 9 | 3.6 |
|  | 258 | 4.33 | 2.20 | 655 | 6 | 3.4 |
|  | 385 | 4.29 | 2.18 | 574 | 20 | 3.3 |
|  | 559 | 4.02 | 1.63 | 630 | 2 | 3.4 |
|  | 686 | 4.45 | 2.46 | 589 | 3 | 2.8 |
| 9051 | 150 | 4.72 | 2.46 | 412 | 2 | 5.1 |
| 9052 | 75 | 2.86 | 1.69 | 686 | 7 | 6.5 |
|  | 143 | 3.59 | 1.69 | 839 | 5 | 5.5 |
|  | 325 | 2.18 | 1.38 | 823 | 4 | 5.9 |
|  | 523 | 3.14 | 2.71 | 777 | 15 | 5.3 |
| 9053 | 72 | 4.08 | 1.69 | 1608 | 6 | 4.1 |
| 9054 | 153 | 3.80 | 1.69 | 601 | 3 | 5.3 |
| 9056 | 45 | 4.38 | 2.54 | 518 | 22 | 5.5 |
|  | 89 | 4.16 | 2.80 | 522 | 9 | 4.6 |
|  | 237 | 3.93 | 2.49 | 567 | 9 | 3.8 |
| 9060 | 133 | 4.69 | 2.88 | 584 | 6 | 5.2 |
| 9064 | 52 | 3.95 | 2.56 | 798 | 11 | 5.8 |
| 9065 | 225 | 4.46 | 2.64 | 441 | 2 | 3.0 |
| 9067 | 54 | 4.17 | 1.88 | 598 | 1 | 2.4 |
|  | 114 | 3.16 | 1.69 | 951 | 2 | 2.5 |
|  | 259 | 3.31 | 1.69 | 590 | 2 | 2.2 |
|  | 432 | 4.21 | 2.18 | 724 | 0 | 3.5 |
| 9069 | 136 | 5.09 | 2.77 | 452 | 12 | 4.0 |
|  | 242 | 4.97 | 2.68 | 472 | 25 | 3.7 |
| 9070 | 316 | 3.28 | 1.69 | 377 | 1 | 2.9 |
|  | 360 | 3.52 | 1.90 | 386 | 4 | 2.8 |
|  | 442 | 3.59 | 2.12 | 323 | 3 | 2.9 |
| 9072 | 250 | 4.90 | 2.99 | 740 | 1 | 3.2 |
| 9078 | 127 | 3.90 | 1.69 | 493 | 3 | 9.0 |
| 9079 | 127 | 4.33 | 2.79 | 641 | 17 | 7.3 |
| 9084 | 61 | 4.60 | 2.61 | 858 | 5 | 3.4 |
|  | 110 | 3.74 | 1.86 | 627 | 12 | 4.3 |
| 9085 | 139 | 4.43 | 2.21 | 590 | 4 | 4.1 |
| 9090 | 333 | 4.37 | 2.83 | 384 | 7 | 3.0 |
| 9091 | 78 | 4.80 | 1.80 | 657 | 3 | 8.7 |
|  | 132 | 4.89 | 1.78 | 557 | 3 | 10.5 |
| 9094 | 20 | 5.01 | 1.74 | 617 | 3 | 3.8 |
| 9095 | 77 | 5.57 | 2.30 | 772 | 1 | 4.5 |
| 9099 | 100 | 4.63 | 2.17 | 259 | 3 | 4.9 |

^a^Time point(s) beyond 2 years p.i. analyzed for subject 9018 and 9040 were not included in any overall population analyses.

^b^Estimated.

^c,d^VL, viral load; HIV-1 RNA (log_10_ copies/ml).

^e^Cells/μl.

^f^CSF white blood cell (WBC) count, cell/μl.

^g^CSF/plasma albumin ratio.
