# Supplementary material for: Compartmentalized Replication of R5 T Cell-Tropic HIV-1 in the Central Nervous System Early in the Course of Infection
Source: PLoS Pathog. 2015 Mar 26;11(3):e1004720. doi: 10.1371/journal.ppat.1004720 (PMC4374811; doi:10.1371/journal.ppat.1004720)
Supplement: S2 Table — (DOCX) [file ppat.1004720.s005.docx]

**S2 Table. Env glycosylation site analysis of compartmentalized subjects.**

| Subject ID | C/P^a^ | Env Glycosylation Site Position^b^ | | | | | | | | | | | | Mean Gly. Freq.^c^ | *P* Value^d^ |
| --- | --- | --- | --- | --- | --- | --- | --- | --- | --- | --- | --- | --- | --- | --- | --- |
| 7146 | C |  |  |  |  |  |  |  | 224 | 247 | 286 |  |  | 25 | 0.0001 |
|  | P |  |  | 13 |  | 17 |  | 67 |  |  |  | 345 |  | 23 |  |
| 9018 | C |  |  | 13 |  |  |  |  |  |  |  |  |  | 21 | <0.0001 |
|  | P | 5 | 9 |  | 14 |  | 56 |  |  |  |  |  |  | 23 |  |
| 9021 | C |  |  |  |  |  |  |  |  |  |  |  |  | 27 | 0.8307 |
|  | P |  |  |  |  |  |  |  |  |  |  |  |  | 27 |  |
| 9040 | C |  |  |  |  |  |  |  |  |  |  |  |  | 26 | 0.0003 |
|  | P |  |  |  |  |  |  |  |  |  |  |  |  | 25 |  |
| 9096 | C |  |  |  |  |  |  |  |  |  |  |  | 460 | 32 | 0.5041 |
|  | P |  |  |  |  |  |  |  |  |  |  |  |  | 32 |  |

^a^Compartmentalized (C) or plasma (P) viral population

^b^Differences in Env glycosylation site positions comparing blood and CSF. No consistent differences in glycosylation sites observed between subjects.

^c^Mean glycosylation site frequency

^d^t test comparing glycosylation site frequencies of compartmentalized and plasma viral populations
