# Supplementary material for: Compartmentalized Replication of R5 T Cell-Tropic HIV-1 in the Central Nervous System Early in the Course of Infection
Source: PLoS Pathog. 2015 Mar 26;11(3):e1004720. doi: 10.1371/journal.ppat.1004720 (PMC4374811; doi:10.1371/journal.ppat.1004720)
Supplement: S3 Table — (DOCX) [file ppat.1004720.s006.docx]

**S3 Table. Selective analysis of amino acid differences between compartmentalized and plasma populations in subjects analyzed for entry tropism in MDMs.**

| Patient | Tropism | 153^a^ | 167^a^ | 178^b^ | 188^b^ | 238^c^ | 240^c^ | 283^d,e^ | 308^f^ | 317^e^ | 326^c^ | 362^g^ | 363^g^ | 364^g^ | 373^g^ | 386^g,h,i^ | 396^i^ |
| --- | --- | --- | --- | --- | --- | --- | --- | --- | --- | --- | --- | --- | --- | --- | --- | --- | --- |
| Consensus (sub. B) | T-tropic | E | D | K | N | P | T | T | H | F | I | N | Q | S | M | N* | N* |
| Proposed mutation | M-tropic | G | N | E | (T→) N | K | K | N | P | L | (M→) I | K | P | (P→) S | K | X | X |
| 9018 T1 | T-tropic | E | D | K | T | P | T | T | N | F | I | N | H | S | M | N | -* |
| 9018 T1 | M-tropic | E | D | K | N | P | T | T | R* | F | I | N | S | S | M | N | -* |
| 9021 T2 | T-tropic | E | -* | K | S | L | K | T | -* | F | I | Q | P | P | M | N | N |
| 9021 T2 | M-tropic | E | -* | K | S | L | K | T | -* | F | I | Q | P | P | M | N | N |
| 9040 T1 | T-tropic | E | D | K | N | S | T | I | H | F | I | N | H | S | M | N | N |
| 9040 T1 | M-tropic | E | D | K | N | S | T | I | H | F | I | N | H | S | M | N | N |
| 9040 T2 | T-tropic | E | D | K | N | S | T | I | H | F | I | N | H | S | M | N | N |
| 9040 T2 | M-tropic | E | D | K | N | S | T | I | H | F | I | N | H | S | M | N | N |
| 9096 T1 | T-tropic | E | D | R | -* | P | K | T | P/H | F | I | K | P | P | M | N | E |
| 9096 T1 | M-tropic | E | N | R | -* | P | K | T | P | F | I | K | P | P | M | N | K |
| 9018 T4 | T-tropic | E | D | K | D | P | T | T | H | F | I | N | H | S | M | D/N | -* |
| 9018 T4 | M-tropic | E | D | K | D | P | T | T | N | F | I | N | H | S | M | N | -* |
| 9018 T5 | T-tropic | A | D | K | D | P | T | T | H | F | I | N | H | S | M | N/D | -* |
| 9018 T5 | M-tropic | D | D | K | D | P | T | T | N | F | I | N | H | S | M | D | -* |
| 9096 T2 | T-tropic | E | D | R | R | P | K | T | H | F | I | K | P | P | M | N | E |
| 9096 T2 | M-tropic | E | D | R | R | P | K | T | P | F | I | K | P | P | M | N | E |
| 9040 T3 | T-tropic | E | D | K | N | P/S | T | I | H | F | I/T | N | H | S | M | N | N |
| 9040 T3 | M-tropic | E | D | K | N | S | T | I | H | F | I | N | H | S | M | N | N |
| 9040 T4 | T-tropic | E | D | K | N | P/S | T | I | H | F | T/V/I | N | H | S | M | N | N |
| 9040 T4 | M-tropic | E | D | K | N | P/S | T | I | H | F | I | N | H | S | M | N | N |

^a^Musich et al. J Virol. 85:2397, 2011.

^b^Walter et al. J Virol. 79:4828, 2005.

^c^Cashin et al. J Virol. 85:10699, 2011.

^d^Dunfee et al. Proc Natl Acad Sci USA. 103:15160, 2006.

^e^Duenas-Decamp et al. J Virol. 83:2575, 2009.

^f^Thomas et al. Virology. 360:105, 2007.

^g^Duenas-Decamp et al. J Virol. 82:5807, 2008.

^h^Dunfee et al. Virology. 361:222, 2007.

^i^Ouyang et al. J Neurovirol. 20:332, 2014.
